# Easy access to heterobimetallic complexes for medical imaging applications via microwave-enhanced cycloaddition

**DOI:** 10.3762/bjoc.11.239

**Published:** 2015-11-17

**Authors:** Nicolas Desbois, Sandrine Pacquelet, Adrien Dubois, Clément Michelin, Claude P Gros

**Affiliations:** 1 Université de Bourgogne Franche-Comté, ICMUB (UMR CNRS 6302), 9 Avenue Alain Savary, BP 47870, 21078 Dijon Cedex, France

**Keywords:** click chemistry, corrole, DOTA, microwave, NOTA, porphyrin

## Abstract

The Cu(I)-catalysed Huisgen cycloaddition, known as “click” reaction, has been applied to the synthesis of a range of triazole-linked porphyrin/corrole to DOTA/NOTA derivatives. Microwave irradiation significantly accelerates the reaction. The synthesis of heterobimetallic complexes was easily achieved in up to 60% isolated yield. Heterobimetallic complexes were easily prepared as potential MRI/PET (SPECT) bimodal contrast agents incorporating one metal (Mn, Gd) for the enhancement of contrast for MRI applications and one “cold” metal (Cu, Ga, In) for future radionuclear imaging applications. Preliminary relaxivity measurements showed that the reported complexes are promising contrast agents (CA) in MRI.

## Introduction

Magnetic resonance imaging (MRI), positron emission tomography (PET) or single photon emission computed tomography (SPECT) are actually the most commonly used imaging modalities. MRI provides high-resolution (at the submillimeter level), but is limited by its low sensitivity. Conversely, PET and SPECT imaging are more sensitive methods but they both suffer from low anatomical resolution. As one single modality is usually not sufficient to obtain all the necessary information, multimodal imaging appears to be a promising solution. The advantages of one technique can easily be combined with the advantages of another one, whilst reducing, at the same time, the disadvantages of both. PET and MRI are largely complementary techniques and combination of both would certainly lead to a ‘marriage of convenience’ [1].

**Figure 1:**
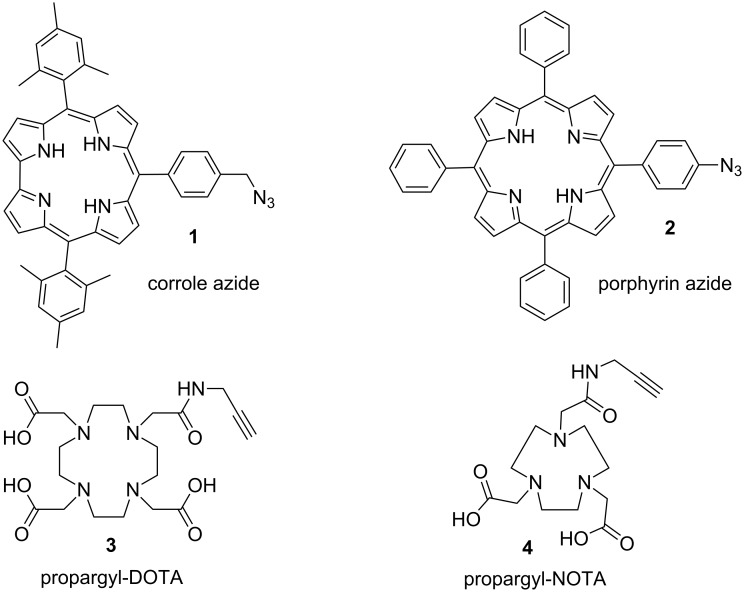
Selected ligands for the copper(I)-catalyzed Huisgen cycloaddition.

Our group previously reported the synthesis of porphyrin-DOTA-like scaffolds for multimodal imaging [2–3]. We were interested in heterobimetallic complexes incorporating both gadolinium and copper atoms. We now want to report new multimodal porphyrinoids-DOTA-like agents incorporating different metal ions, e.g., Mn, Ga, In, etc. of potential interest in medical imaging. Many examples of bimodal agents have been recently reported in the literature [4–6]. However, most of the bimodal agents were prepared for a specific application. To our part, our idea was to develop a toolbox of different dyads for further coordination of different types of metals, the easy and fast assembly of two polyazamacrocycles providing easy access to new bimodal probes. Depending upon the desired application, it could allow choosing one paramagnetic metal for MRI and one radiometal for application in PET. The ‘Huisgen’ Cu(I)-catalyzed cycloaddition appears to be a good answer to access to such bimodal agents [7]. For example, Caravan and co-workers have described an MRI-PET agent using a copper(I)-catalyzed Huisgen cycloaddition [8]. To prepare new multimodal agents, we have chosen two porphyrinoid derivatives that are easy to prepare in only a few steps: an azidocorrole **1** [9] and an azidoporphyrin **2** [10] (Figure 1).

Porphyrins and corroles display interesting properties in terms of metal complexation of, e.g., transition metals. We have selected two commercial available alkynes e.g. a propargyl-DOTA-tris(*t*-Bu)ester and a propargyl-NOTA(*t*-Bu)_2_. Indeed, 1,4,7,10-tetraazacyclododecane-1,4,7,10-tetraacetic acid (DOTA, **3**) and 1,4,7-triazacyclononane-1,4,7-triacetic acid (NOTA, **4**) are well-known to form highly stable Gd(III) and Ga(III) complexes and they are intensely studied as chelates for many imaging modalities.

To further demonstrate the versatility of the selected chelating agents, we have chosen two metals for MRI applications (Mn and Gd) and three metals (“cold” models of radionuclide) for possible nuclear imaging applications (e.g., Cu, Ga, In).

**Gadolinium.** There have been many examples in recent years of targeted contrast agents prepared via the use of an amide side arm Gd–DOTA [11]. In the case of porphyrin derivatives, gadolinium complexes are unstable (a fast demetallation reaction is usually observed) in many common solvents like toluene, methylene chloride, chloroform or methanol [12] thus MRI applications of Gd-porphyrins are difficult to envisage.

**Manganese.** The design of gadolinium complex did not fully eliminate the risk of in vivo release and accumulation which is a common problem implicated in nephrogenic systemic fibrosis. A possible solution is to replace Gd(III) with a more biocompatible metal species, ideally displaying higher relaxivity at high fields. Manganese is a promising candidate with limited toxicity at low concentrations. Up to now and so far to our knowledge, no Mn–DOTA/NOTA complexes have found application as contrast agents for MRI. Indeed, manganese complexes of DOTA/NOTA macrocycles are thermodynamically less stable than other transition metal ions or Gd^3+^ analogues [13]. Over the years and to overcome this problem, a variety of Mn(III) porphyrins have been prepared and investigated for their potential use as MRI contrast agent [14–15].

**Copper-64.** Despite the emerging recognition of ^64^Cu as a suitable radioisotope in positron emitting tomography (PET) imaging (*t*_1/2_ = 12.7 h, β^+^: 17.4%, *E*_β+max_ = 656 keV; β^−^: 39%, *E*_β-max_ = 573 keV), there is actually only few examples of the association of ^64^Cu^2+^-labeled NOTA/DOTA [16–17] and porphyrins as PET probes. Cu–NOTA is significantly more inert in acidic conditions compared to Cu–DOTA, which usually decomplexed within few minutes. ^64^Cu^2+^-NOTA is considered as better PET agent, with regard to its in vivo stability [18]. Porphyrins are also excellent macrocyclic ligands forming highly stable transition metal complexes making them efficient delivery vehicles for radioisotopes. The radioactive ^64^Cu^2+^-porphyrin is known to be extremely stable. Shi et al. [19] have described an easy and efficient radiolabeling of the porphyrin macroring with ^64^Cu. Mukai et al. [20] have reported the successful synthesis of ^64^Cu-chelated porphyrin photosensitizers and a tumor targeting peptide. To the best of our knowledge, no previous example of the preparation of radioligand ^64^Cu corrole has been so far reported. Due to their easy copper insertion [21], corroles can be considered as really good candidates for the complexation of ^64^Cu. In solution, free-base corroles are more sensitive than the analog porphyrins and may decompose to some open biliverdin-type structures upon air and light exposure. However, metallocorroles are generally more stable than their free-base form [21].

**Gallium-68.** NOTA [22] and DOTA [23] have been reported to form a stable complex with gallium(III) and those two ligands are widely used in the preparation of ^68^Ga-based PET probes. Advantageously, Ga complexes of porphyrins and corroles are known to be very stable allowing possible PET imaging applications [24].

**Indium-111.** One major isotope used within DOTA–SPECT agents is ^111^In (displaying a half-live of 67 hours). It should be noted that the well-established ^111^In–DOTA is in vivo robust [25] and many articles have been reported in the literature highly supporting the potential convenient utility of radiolabeled ^111^In–NOTA [26]. Unlike the corrole macrocycle, indium(III) porphyrins can be prepared [27].

**Scheme 1:**
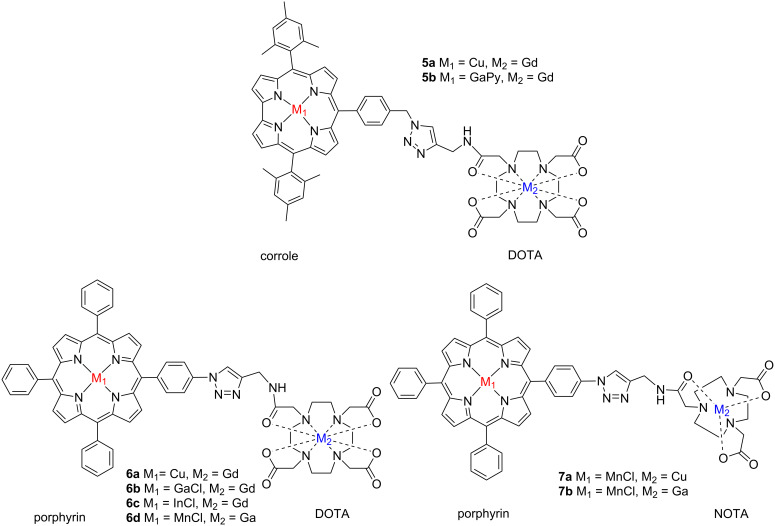
Structure of different bimetallic complexes **5–7**.

We wish to report herein (Scheme 1) the convenient synthesis of various multimodal ligands.

Those complexes, incorporating one porphyrin or one corrole moiety on one side and one DOTA or NOTA-like macrocycle on the other side, have been easily linked by microwave azide–alkyne 1,3-dipolar cycloaddition.

The preliminary relaxivity study of these heterobimetallic complexes was also investigated for potential MRI applications.

## Results and Discussion

We first wish to report a “library” of azido and alkyne derivatives to further interconnect by microwave-assisted click reaction.

### Azidocorroles/porphyrins

**Scheme 2:**
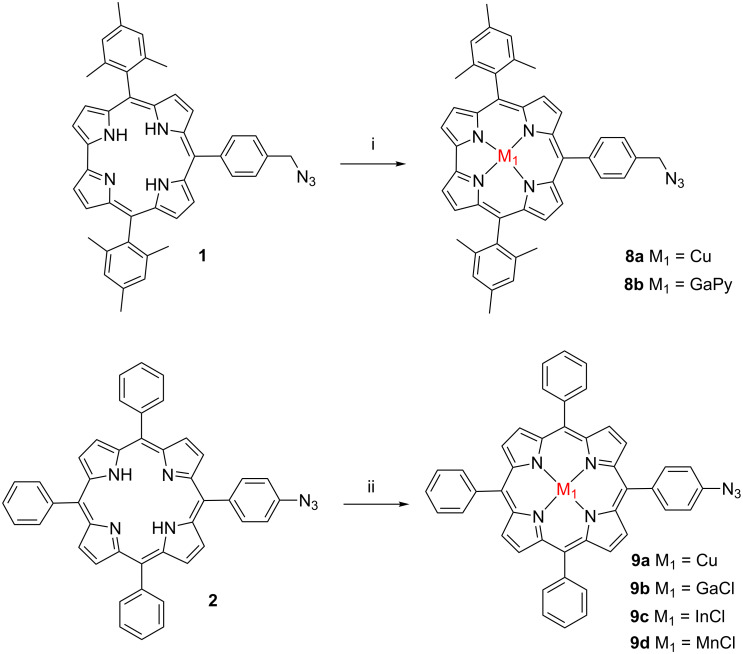
Synthesis of **8a**,**b** and **9a–d**. (i) for **8a**: THF, N_2_, Cu(OAc)_2_·H_2_O, rt 15 min; for **8b**: GaCl_3_ 0.114 M in dry pyridine, reflux 1.5 h. (ii) for **9a**: CHCl_3_/MeOH, Cu(OAc)_2_·H_2_O, reflux 12 h; for **9b**: GaCl_3_, AcOH, AcONa, reflux overnight; for **9c**: InCl_3_, AcOH, AcONa, reflux 18 h; for **9d**: MnCl_2_·4H_2_O, benzonitrile, reflux 1.5 h.

Azidocorrole **1** [9] was obtained by the condensation of the corresponding *meso*-mesityl-substituted dipyrromethane with 4-azidobenzaldehyde in the presence of a catalytic amount of trifluoroacetic acid, followed by oxidation under the action of DDQ. Cu and Ga complexes were prepared by the strategy outlined in Scheme 2.

The insertion of Cu into corrole **1** was easily achieved with Cu(OAc)_2_·H_2_O in THF during 15 min. To obtain the gallium corrole **8b**, free-base azidocorrole **1** was dissolved in a solution of GaCl_3_ in pyridine and then refluxed for 1.5 h as previously described [28]. Corroles **8a** and **8b** were prepared and characterized by ^1^H NMR, HRMS and UV–vis absorption spectroscopy.

Different metal ions were introduced inside the porphyrin core **2**, namely copper, gallium, indium and manganese. For the synthesis of copper porphyrin **9a**, compound **2** [10] was reacted with Cu(OAc)_2_. The gallium complex of porphyrin **2** was prepared starting from GaCl_3_ in refluxing AcOH. The insertion of indium into porphyrin **2** was achieved by heating with InCl_3_ salt in AcOH. Finally, manganese chloride and **2** were dissolved in benzonitrile and then refluxed to yield **9d**. The target metalated porphyrins **9a–d** were isolated in moderate to good yields ranging from 51 to 82% and were fully characterized by ^1^H NMR, MS (MALDI–TOF) and HRMS (ESI) spectrometry.

### Alkynyl DOTA/NOTA

**Scheme 3:**
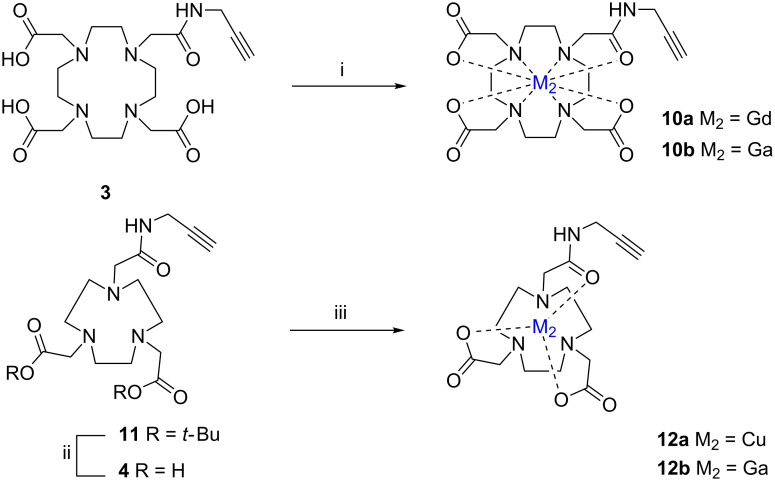
Synthesis of **10a**,**b** and **12a**,**b**. (i) For **10a**: Milli-Q water, Gd(NO_3_)_3_·5H_2_O, 50 °C, 17 h, pH 8.0; for **10b**: Ga(NO_3_)_3_, NH_4_OAc, pH 3, rt, 4 h; ii) TFA, overnight; (iii) for **12a**: H_2_O, Cu(ClO_4_)_2_·6H_2_O, pH 8, rt, 1 h; for **12b**: H_2_O, Ga(NO_3_)_3_, AcONa 0.5 M, pH 3, reflux 30 min.

In a second step, the successful preparation of azidocorroles and porphyrins allows us to study their reactivity with different alkynes. To this end, a series of DOTA/NOTA complexes carrying alkyne reactive units were synthesized (Scheme 3).

DOTA carrying a propargylamido functionality **3** was prepared according to a literature procedure starting from commercially available propargylamido-DOTA-tris(*t*-Bu)ester [29]. Complexation of **3** with a lanthanide was previously described by Borbas et al. [30]. Gd(DOTA) complex **10a**, containing a pendant terminal alkyne group, was prepared by variation of a known procedure [29,31], as shown in Scheme 3. The complex was obtained by precipitation from a concentrated methanolic solution by slow addition of diethyl ether. Compound **3** was reacted with Ga(NO_3_)_3_ to lead to **10b** in 94% yield after flash chromatography. The IR spectrum of **10b** exhibits the characteristic band of the carbonyl stretching vibration from 1722 cm^−1^ to 1656 cm^−1^, which clearly demonstrates the complexation of Ga^3+^ ions with the carboxylic acid groups of ligand **3**.

Another macrocycle commonly used in medical imaging studies is the NOTA ligand, which is usually preferred to DOTA to chelate e.g. Cu^2+^. Propargyl-NOTA(*t*-Bu)_2_ **11** (commercially available) was first dissolved in trifluoroacetic acid (TFA) and stirred at room temperature overnight. A brown oil was obtained upon concentration. The residue was then purified by flash chromatography. Chelation of Cu^2+^ with propargyl NOTA **4** was performed in aqueous solution by Cu(ClO_4_)_2_·6H_2_O. Purification of Cu propargyl NOTA was performed by precipitation in diethyl ether in 94% yield. To prepare the gallium complex **12b**, propargyl NOTA **4** and Ga(NO_3_)_3_ were dissolved, at pH 3, in water and then refluxed for 30 min. The pH of the reaction was adjusted (AcONa 0.5 M), in each case, to optimize the complexation kinetic (e.g*.,* pH fix at 7 for Cu^2+^ and pH fix at 3 for Ga^3+^). The isolated copper and gallium NOTA derivatives **12a**,**b** were characterized by high-resolution mass spectrometry.

### Scope of the cycloaddition reaction

Over the past decade, click chemistry has been applied to the synthesis of a wide variety of radiolabeled imaging agents with increasing frequency [7,32]. A methodology for the click reaction of azidocorrole or porphyrin has been recently developed in our laboratory [28,33]. We thus used similar conditions. The reactions were carried out in DMF, using excess of the alkyne derivative in the presence of the azido counterpart, CuI and DIPEA. A very slow progress of the reaction was observed by TLC during the synthesis of **5a** at room temperature. Thus the reaction mixture was heated to 50 °C. Consumption of the azidocorrole **8a** (starting default reactant) was observed by TLC, in 4 hours. Unfortunately, increasing the temperature did not speed up the kinetic of the reaction (e.g. decrease the reaction time). Instead, degradation of corrole **8a** was observed upon time. An optimization of the reaction conditions was thus carried out to find reaction conditions as much as possible compatible with the future use of radioactive isotopes possessing short life times (e.g*.* Cu^2+^ and In^3+^). Microwave irradiation, known to accelerate the polarization of the starting materials to promote the reactions, was investigated. A mixture of azidocorrole **8a**, Gd propargyl DOTA **10a**, CuI, DIPEA and DMF were irradiated in a quartz vessel using a microwave oven at 60 W for 30 min. After evaporation of DMF and DIPEA, the obtained precipitate was washed with dichloromethane (to remove any starting azido corrole **8a**), washed with water (to remove excess propargyl DOTA **10a**) then washed with ammonia aqueous solution 5.5% (to remove Cu(NH_3_)_2_ complex). The successful formation of the Cu corrole/Gd DOTA complex **5a** was confirmed by HRMS (ESI) mass spectrometry by the presence of the pseudo-molecular ion peak [M + H]^+^ at 1322.3695 (4.9 ppm deviation with respected to calculated mass). The same procedure was adopted for the preparation of bimetallic compounds **5**–**7** as shown in Table 1.

**Table 1:** Scope of the cycloaddition reaction.^a^

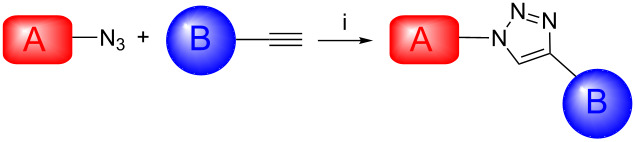			

Compound	**A**	**B**	Yield

**5a**	**8a(Cu)**	**10a(Gd)**	80%
**5b**	**8b(GaPy)**	**10a(Gd)**	77%
**6a**	**9a(Cu)**	**10a(Gd)**	27%
**6b**	**9b(GaCl)**	**10a(Gd)**	44%
**6c**	**9c(InCl)**	**10a(Gd)**	26%
**6d**	**9d(MnCl)**	**10b(Ga)**	76%
**7a**	**9d(MnCl)**	**12a(Cu)**	59%
**7b**	**9d(MnCl)**	**12b(Ga)**	43%

^a^Reaction conditions: corrole/porphyrin complex: 1 equiv, coupling partner: DOTA/NOTA 1.5 equiv; CuI: 3 equiv, DIPEA: 5 equiv, DMF, microwave heating.

The reaction conditions used for all the reported examples were identical to those for **5a**, and the yields were from moderate to good. All the final products were characterized by HRMS, UV–vis and IR spectroscopy.

### Relaxivity measurements

The relaxivity measurements is a complementary method for the complete characterization of a paramagnetic complex. As described by Shu [34], the ability of proton relaxation enhancement of a paramagnetic compound is expressed by the term relaxivity r_i_ as denoted in Equation 1

[1]
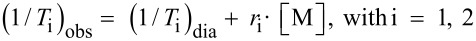


where 1/*T*_1_ and 1/*T*_2_ are, respectively, the longitudinal and transverse relaxation rates, [M] is the concentration of the paramagnetic species, (1/*T*_i_)_obs_ and (1/*T*_i_)_dia_ are defined as the observed relaxation rate of water proton in the presence and in the absence of the paramagnetic species, respectively. The relaxivities of compound **9d**, **11a** and **5–7** were measured at 0.47 T and 40 °C. As shown in Table 2, Mn azidoporphyrin **9d** and Gd propargyl-DOTA **10a** exhibited similar relaxivities than DOTAREM contrast agent widely used in clinical practice (*r*_1_ = 3.5 mM^−1^ s^−1^ at 20 MHz, 37 °C) [35]. In contrast, most of bimodal complexes **5–7** exhibited higher relaxivities than DOTAREM. The molecular weight of here described complexes is significantly higher than the commercially available DOTA derivatives, inducing a slower molecular tumbling known to have a key effect on the increase of the relaxivity [36].

**Table 2:** Relaxivity measurements.

Gd/Mn Complex	Relaxivity *r*_1_^a^ (mM^−1^ s^−1^)

**9d**	2.0
**10a**	2.2
**5a**	9.2
**5b**	6.3
**6b**	8.2
**6c**	11.6
**7a**	3.4
**7b**	5.7

^a^20 MHz, 0.47 T, 40 °C.

## Conclusion

Recent synthetic trategies using click chemistry have shown significant advantages over traditional procedures for the modular, rapid, clean, and efficient synthesis of potential radiopharmaceuticals. In this regard, click chemistry has already begun to revolutionize radiopharmaceutical chemistry. The method reported herein provided convenient and rapid access to bimetallic building blocks, opening the door for a wide range of applications in medical imaging.

In some cases, the requirement of a metal catalyst can be a complication. Therefore, we actually focus our researches on the development of catalyst-free click reactions using, e.g., cyclooctyne DOTA derivatives.

## Supporting Information

File 1 Materials, methods and experimental procedures. ^1^H NMR spectra of **8a**, **9b,c**, **4**, **12b**. HRMS spectra of **8a**, **9a–d**, **10b**, **4**, **12a,b**, **5a,b**, **6a,d**, **7a,b**.
