# Supplementary material for: Easy access to heterobimetallic complexes for medical imaging applications via microwave-enhanced cycloaddition
Source: Beilstein J Org Chem. 2015 Nov 17;11:2202–8. doi: 10.3762/bjoc.11.239 (PMC4660971; doi:10.3762/bjoc.11.239)
Supplement: File 1 — Materials, methods and experimental procedures. 1H NMR spectra of 8a, 9b,c, 4, 12b. HRMS spectra of 8a, 9a–d, 10b, 4, 12a,b, 5a,b, 6a,d, 7a,b. [file Beilstein_J_Org_Chem-11-2202-s001.pdf]

Supporting Information

for

Easy access to heterobimetallic complexes for medical imaging applications via microwave-enhanced cycloaddition

Nicolas Desbois, Sandrine Pacquelet, Adrien Dubois, Clément Michelin and Claude P. Gros*

Address: Université de Bourgogne Franche-Comté, ICMUB (UMR CNRS 6302), 9 Avenue Alain Savary, BP 47870, 21078 Dijon Cedex, France

Email: Claude P. Gros - claude.gros@u-bourgogne.fr
*Corresponding author

Materials, methods and experimental procedures. ^{1}H NMR spectra of 8a, 9b,c, 4, 12b. HRMS spectra of 8a, 9a–d, 10b, 4, 12a,b, 5a,b, 6a,d, 7a,b.

Figure S1. ^{1}H NMR spectrum of **8a** in $CDCl_3$ s10
Figure S2. MS (MALDI TOF) and HRMS (ESI) mass spectra of **8a** s11
Figure S3. HRMS (ESI) mass spectra of **9a** s12
Figure S4. ^{1}H NMR spectrum of **9b** in $CDCl_3$ s13
Figure S5. HRMS (ESI) mass spectrum of **9b** s14
Figure S6. ^{1}H NMR spectrum of **9c** in $CDCl_3$ s15
Figure S7. HRMS (ESI) mass spectrum of **9c** s16
Figure S8. HRMS (ESI) mass spectrum of **9d** s17
Figure S9. MS and HRMS (ESI) mass spectra of **10b** s18
Figure S10. ^{1}H NMR spectrum of **4** in MeOD s19
Figure S11. MS and HRMS (ESI) mass spectra of **4** s20
Figure S12. HRMS (ESI) mass spectrum of **12a** s21
Figure S13. ^{1}H NMR spectrum of **12b** in D_2O s22
Figure S14. HRMS (ESI) mass spectrum of **12b** s23
Figure S15. MS (MALDI TOF) and HRMS (ESI) mass spectra of **5a** s24
Figure S16. MS and HRMS (ESI) mass spectra of **5b** s25
Figure S17. HRMS (ESI) mass spectrum of **6a** s26
Figure S18. HRMS (ESI) mass spectrum of **6b** s27
Figure S19. HRMS (ESI) mass spectrum of **6c** s28
Figure S20. HRMS (ESI) mass spectrum of **6d** s29
Figure S21. HRMS (ESI) mass spectrum of **7a** s30
Figure S22. HRMS (ESI) mass spectrum of **7b** s31

Experimental

Physicochemical characterization of compounds

^{1}H NMR spectra were recorded on a Bruker Avance II 300 (300 MHz) or on a Bruker Avance DRX 600 (600 MHz) spectrometer; chemical shifts are expressed in ppm relative to chloroform (7.26 ppm), methanol (3.31 ppm) or D_2O (4.79 ppm). Mass spectra and accurate mass measurements (HRMS) were obtained on a Bruker Daltonics Ultraflex II spectrometer in the MALDI/TOF reflectron mode using dithranol or 2,5-dihydroxybenzoic acid (DHB) as a matrix or on a LTQ Orbitrap XL (THERMO) instrument in ESI mode. Infrared spectra were recorded on an IR FT BRUKER Vertex 70v. Elemental analyses were performed with an Elemental Analyser Thermo electron Flash EA 1112. Measurements were made at the "the "*Welience, Pôle Chimie Moléculaire de l'Université de Bourgogne* (*WPCM*)". Microwave reactions were carried out in a MicroSYNTH (Milestone) Microwave reactor. UV–vis spectra were recorded on a Varian Cary 1 spectrophotometer.

Relaxivity measurements

The longitudinal relaxation times T_1 were measured at 20 MHz (0.47 T) and at 40 °C on a Bruker Minispec ''mqvar''. Solutions for relaxivity measurements were prepared by dissolving the gadolinium complexes into an H_2O:DMSO (95:5) mixture. The exact Gd(III) ion concentration was determined by emission spectrometry on a Vista AX CCD Simultaneous ICP-AES Varian spectrophotometer. The complexes were mineralized (using HNO_3/H_2O_2, 4 mL/1 mL) by microwave-assisted mineralization before ICP-AES measurements. For each measurement of the T_1 longitudinal relaxation times, three solutions of the complexes at different concentrations were prepared ([Gd] = 0.1, 0.05 and 0.02 mM). T_1 values were measured by the classical

inversion recovery sequence with 10 data points, each solution was incubated at 40 °C for 10 min before measurement. The longitudinal relaxivity (r_1) was determined as the slope of the line of $1/T_1$ versus Gd concentration.

(Cu) azido-corrole complex 8a

. The corrole **1** (100 mg, 15.0 μmol) was dissolved in THF (18 mL), under N_2, and $Cu(OAc)_2{\cdot}H_2O$ (72.0 mg, 36.1 μmol) was added dropwise. The solution was stirred at room temperature shielded from light and under N_2 during 15 min. The solvent was removed under reduced pressure. Column chromatography of the crude product using dichloromethane / heptane (1/1, v/v) over silica gel afforded 45.0 mg (41%) of the title compound. ^1H NMR (300 MHz, $CDCl_3$) δ (ppm): 2.34 (s, 12H, CH_3), 2.68 (s, 6H, CH_3), 4.75 (s, 2H, CH_2), 7.30 (s, 4H, H_{Mes}), 7.43 (m, 4H, H_β), 7.63 (m, 2H, H_β), 7.72 (d, 2H, J = 7.5 Hz, H_{Ph}), 7.89 (d, 2H, J = 7.5 Hz, H_{Ph}), 8.24 (m, 2H, H_β). UV/Vis (DCM): λ_{max} (nm) (ε x 10^{-3} L mol^{-1} cm^{-1}) = 414 (128), 538 (14). MS (MALDI-TOF) *m/z* = 724.99 $[M]^{+\cdot}$, 725.23 calcd for $C_{44}H_{36}CuN_7$, *m/z* = 683.00 $[M-N_3]^+$, 683.23 calcd for $C_{44}H_{36}CuN_4$. HRMS (ESI) *m/z* = 725.2282 $[M]^{+\cdot}$, 725.2323 calcd for $C_{44}H_{36}CuN_7$. Anal. Calc. for $C_{44}H_{36}CuN_7.3H_2O.2CH_2Cl_2$ requires: C, 58.14; H, 4.88; N, 10.32. Found: C, 60.41; H, 4.77; N, 8.90.

(Ga) azido-corrole complex 8b. This product was prepared according to the literature [1]. Anal. Calc. for $C_{49}H_{41}GaN_8.0.5C_5H_{12}$ requires: C, 72.51; H, 5.09; N, 13.81. Found: C, 72.97; H, 5.59; N, 13.22.

(Cu) azido-porphyrin complex 9a. N_3-$TPPH_2$ **2** (100 mg, 0.152 mmol) was dissolved in chloroform (15 mL) and a solution containing $Cu(OAc)_2{\cdot}H_2O$ (277 mg, 1.39 mmol) in 15 mL of methanol was added dropwise at room temperature. Then the mixture was allowed to reflux for 12 h. The reaction mixture was washed with deionized water. The chloroform layer was separated, dried over $MgSO_4$ and

evaporated to dryness under vacuum. The crude product was purified by column chromatography (silica gel, eluent CH_2Cl_2) to give the title compound in 82% (89.0 mg, 0.124 mmol). UV/Vis (DCM): λ_{max} (nm) (ε x 10^{-3} L mol^{-1} cm^{-1}) = 415 (421), 539 (16), 575 (1). HRMS (ESI) m/z = 716.1642 $[M]^{+\cdot}$, 716.1628 calcd for $C_{44}H_{27}CuN_7$.

(Ga) azido-porphyrin complex 9b. N_3-TPPH$_2$ **2** (40.0 mg, 60.1 µmol), anhydrous sodium acetate (122 mg, 1.49 mmol), and $GaCl_3$ (29.6 mg, 0.168 mmol) were added to glacial acetic acid (11 mL). The mixture was stirred and refluxed overnight under N_2. The reaction mixture was allowed to cool to room temperature. After removal of acetic acid, the product was extracted with $CHCl_3$, dried over $MgSO_4$ and evaporated to dryness under vacuum. The crude product was purified by column chromatography (silica gel, eluent CH_2Cl_2/MeOH, 95/5, v/v) to give the title compound in 62% (29 mg, 0.038 mmol). ^{1}H NMR (300 MHz, $CDCl_3$) δ (ppm): 7.44 (d, 2H, *J* = 8.5 Hz, H_{PhN3}), 7.79 (m, 9H, H_{Ph}), 8.21 (m, 8H, $H_{Ph,}$ H_{PhN3}), 9.10 (m, 8H, H_β). UV/Vis (DCM): λ_{max} (nm) (ε x 10^{-3} L mol^{-1} cm^{-1}) = 420 (315), 550 (12), 591 (2). HRMS (ESI) m/z = 722.1570 $[M\text{-}Cl]^+$, 722.1578 calcd for $C_{44}H_{27}GaN_7$.

(In) azido-porphyrin complex 9c. N_3-TPPH$_2$ **2** (40.0 mg, 60.1 µmol), anhydrous sodium acetate (122 mg, 1.49 mmol), and $InCl_3$ (134.2 mg, 0.61 mmol) were added to glacial acetic acid (18 mL). The mixture was stirred and refluxed during 18 h. The reaction mixture was then allowed to cool to room temperature. After removal of acetic acid, the product was redissolved in CH_2Cl_2. The organic layer was washed with saturated $NaHCO_3$ solution, with brine and dried over $MgSO_4$. CH_2Cl_2 was evaporated to dryness under vacuum. The crude product was purified by column chromatography (silica gel, eluent CH_2Cl_2/MeOH, 98/2, v/v) to give the title compound in 51% (24 mg, 0.030 mmol). ^{1}H NMR (300 MHz, $CDCl_3$) δ (ppm): 7.46 (m, 2H, H_{PhN3}), 7.80 (m, 9H, H_{Ph}), 8.10 (m, 6H, H_{Ph}), 8.38 (m, 2H, H_{PhN3}), 9.05 (m, 8H,

H_β). HRMS (ESI) m/z = 768.1351 $[M-Cl]^+$, 768.1362 calcd for $C_{44}H_{27}InN_7$. UV/Vis (DCM): λ_{max} (nm) (ε x 10^{-3} L mol^{-1} cm^{-1}) = 426 (469), 562 (18), 601 (10).

(Mn) azido-porphyrin complex 9d. N_3-$TPPH_2$ **2** (100 mg, 0.152 mmol) and $MnCl_2.4H_2O$ (91.0 mg, 0.460 mmol) were dissolved in benzonitrile (6 mL). The mixture was allowed to reflux for 1.5 h and then evaporated to dryness under vacuum. The product was then dissolved in dichloromethane (25 mL) and washed with water. The organic layer was separated, dried over $MgSO_4$ and evaporated to dryness under vacuum. The crude product was purified by column chromatography (silica gel, eluent CH_2Cl_2 to CH_2Cl_2/MeOH (97 : 3)) to give the title compound in 55% (60.0 mg, 0.085). MS (MALDI-TOF): m/z = 707.96 $[M-Cl]^{+\cdot}$, 708.17 calcd for $C_{44}H_{27}MnN_7$. HRMS (ESI) m/z = 708.1686 $[M-Cl]^+$, 708.1703 calcd for $C_{44}H_{27}MnN_7$. UV/Vis (MeOH): λ_{max} (nm) (ε x 10^{-3} L mol^{-1} cm^{-1}) = 379 (21), 401 (23), 414 (27), 468 (38), 514 (2), 567 (3), 600 (3).

(Gd) Propargyl-DOTA complex 10a. Compound **3**[2] (160 mg, 0.362 mmol) was dissolved in Milli-Q water (8.0 mL) and the pH was adjusted to 8 with two NaOH aqueous solutions (1 M and 0.1 M). Then a solution of gadolinium(III) nitrate pentahydrate (176 mg, 0.406 mmol) in Milli-Q water (1.0 mL) was added and the contents were heated at 50 °C for 17 h. The pH was periodically checked and adjusted to 8.0 using 0.1 M NaOH aqueous solution. The water was removed by rotary evaporation and the resulting oil was dissolved in a minimal volume of MeOH. Addition of excess diethyl ether produced an off-white solid. The title compound was isolated in 93% yield (207 mg, 0.151 mmol). Experimental data are identical to those in the literature [2].

(Ga) Propargyl-DOTA complex 10b. Compound **3** (100 mg, 0.226 mmol) was dissolved in aqueous ammonium acetate 0.01 M (pH = 4.5). A solution of $Ga(NO_3)_3$

(579 mg, 2.26 mmol) in aqueous ammonium acetate 0.01 M was added (pH 3). The reaction mixture was stirred to room temperature for 4 h. The solvent was removed and the residue was purified by flash column chromatography on C-18 with water and acetonitrile as eluent (100% of water during 5 min to 100% of acetonitrile during 30 min). The title compound was isolated in 94% yield (108 mg, 0.213 mmol). MS (ESI) *m/z* = 508.08 $[M+H]^+$, 508.12 calcd for $C_{19}H_{29}GaN_5O_7$. HRMS (ESI) *m/z* = 508.1314 $[M+H]^+$, 508.1317 calcd for $C_{19}H_{29}GaN_5O_7$. IR: 1656 cm^{-1}.

Propargyl-NOTA 4. Commercially available propargyl-NOTA$(tBu)_2$ (**11**) (106.6 mg, 0.236 mmol) was stirred at room temperature with TFA (7 mL) overnight. The excess of TFA was removed and the residue was purified by flash column chromatography on C-18 with water and acetonitrile as eluent (100% of water during 5 min to 100% of acetonitrile during 30 min). The title compound was isolated in 50% yield (39 mg, 0.115 mmol). ^1H NMR (300 MHz, MeOD) δ (ppm): 2.55 (t, 1H, *J* = 2.4 Hz, CH-alkyne), 2.89 (m, 4H, CH_2), 3.11 (m, 8H, CH_2), 3.53 (s, 2H, CH_2), 3.79 (s, 4H, CH_2), 3.99 (d, 2H, *J* = 2.4 Hz, CH_2-alkyne). MS (ESI) *m/z* = 338.97. $[M-H]^-$, 339.17 calcd for $C_{15}H_{23}N_4O_5$. HRMS (ESI) *m/z* = 341.1804 $[M+H]^+$, 341.1820 calcd for $C_{15}H_{25}N_4O_5$. Anal. $C_{15}H_{24}N_4O_5$.3TFA.CH_3CN requires: C, 38.18; H, 4.18; N, 9.68. Found: C, 37.99; H, 4.29; N, 10.34. IR: 1660 cm^{-1}.

(Cu) Propargyl-NOTA complex 12a. Compound **4** (19.0 mg, 55.8 μmol) and $Cu(ClO_4)_2{\cdot}6H_2O$ (21.0 mg, 56.7 μmol) were dissolved in water. The pH was adjusted to 7 with 1 M NaOH aqueous solution. The reaction mixture was stirred to room temperature for 1 h. The water was removed by rotary evaporation and the resulting oil was dissolved in a minimal volume of MeOH. Addition of excess diethyl ether produced a precipitate. The title compound was isolated in 94% yield (21.0 mg, 52.3 μmol). HRMS (ESI) *m/z* = 424.0782 $[M+Na]^+$, 424.0778 calcd for $C_{15}H_{22}CuN_4NaO_5$.

IR: 1581 cm^{-1}.

(Ga) Propargyl-NOTA complex 12b. Compound **4** (24.3 mg, 71.4 μmol) was added to a solution of $Ga(NO_3)_2$ (36.5 mg, 0.143 mmol) in H_2O (2 mL). An aqueous sodium acetate 0.5 M was added (pH 3) and the reaction mixture was stirred at reflux for 30 min. The solvent was removed and the resulting oil was dissolved in a minimal volume of MeOH. Addition of excess diethyl ether produced an off-white solid. The title compound was isolated in 96% yield (28.0 mg, 68.6 μmol). ^{1}H NMR (300 MHz, D_2O) δ (ppm): 2.84 (t, 1H, *J* = 2.4 Hz, CH-alkyne), 3.27 (m, 8H, CH_2), 3.54 (m, 4H, CH_2), 3.93 (m, 4H, CH_2), 4.30 (s, 2H, CH_2), 4.35 (d, 2H, *J* = 2.4 Hz, CH_2-alkyne). HRMS (ESI) *m/z* = 407.0835 $[M+H]^+$, 407.0841 calcd for $C_{15}H_{22}GaN_4O_5$.

General procedure for the Huisgen cycloaddition reaction. 10-(4-Azidomethylphenyl)-5,15-dimesityl-corrole **8a,b** or porphyrin complexes **9a–d** (20.0 mg), the appropriate DOTA **10a,b** or propargyl-NOTA complex **12a,b** (1.5 equiv), DIPEA (5 equiv) and CuI (3 equiv) were placed in a microwave vial. The solids were suspended in DMF (6 mL) and the vial was sealed. The mixture was heated (60 W) at 50 °C for 30 min. The mixture was then allowed to cool to room temperature. The solvent was removed and the resulting product was precipitated in dichloromethane. The mixture was filtered, washed with dichloromethane, with aqueous ammonia solution (5.5%) then with water. The pure product was collected and dried under reduced pressure.

(Cu) corrole (Gd) DOTA complex 5a. The title compound was isolated as a brown solid in 80% yield (28.7 mg, 21.4 μmol). UV-visible (MeOH): λ_{max} (nm) (ε x 10^{-3} L mol^{-1} cm^{-1}) = 397 (33), 410 (34), 534 (4). MS (MALDI-TOF) *m/z* = 1321.41 $[M-H_2O]^{+\cdot}$, 1321.36 calcd for $C_{66}H_{64}CuGdN_{12}O_7$. HRMS (ESI) *m/z* = 1322.3695 $[M-H_2O+H]^+$, 1322.3636 calcd $C_{66}H_{65}CuGdN_{12}O_7$. IR: 1595 cm^{-1}.

(Ga) corrole (Gd) DOTA complex 5b. The title compound was isolated as a green microcrystalline solid in 77% yield (33.9 mg, 23.8 μmol). UV-visible (MeOH, 0.1% pyridine): λ_{max} (nm) (ε x 10^{-3} L mol^{-1} cm^{-1}) = 413 (40), 530 (3), 568 (3), 613 (4). MS (ESI) *m/z* = 1327.25 [M-Pyridine-H_2O]$^+$, 1327.35 calcd for $C_{63}H_{64}GaGdN_{12}O_7$. HRMS (ESI) *m/z* = 1327.3563 [M-pyridine-H_2O]$^+$, 1327.3517 calcd $C_{66}H_{64}GaGdN_{12}O_7$. IR: 1607 cm^{-1}.

(Cu) porphyrin (Gd) DOTA complex 6a. The title compound was isolated as a purple microcrystalline solid in 27% yield (10.0 mg, 7.51 μmol). UV-visible (MeOH): λ_{max} (nm) (ε x 10^{-3} L mol^{-1} cm^{-1}) = 411 (34), 538 (1). HRMS (ESI) *m/z* = 1313.2943 [M-H_2O+H]$^+$, 1313.2931 calcd $C_{63}H_{56}CuGdN_{12}O_7$. HRMS (ESI) *m/z* = 1335.2745 [M-H_2O+Na]$^+$, 1335.2751 calcd $C_{63}H_{55}CuGdN_{12}O_7Na$. IR: 1578 cm^{-1}.

(Ga) porphyrin (Gd) DOTA complex 6b. The title compound was isolated as a purple microcrystalline solid in 44% yield (15.7 mg, 11.4 μmol). UV-visible (MeOH): λ_{max} (nm) (ε x 10^{-3} L mol^{-1} cm^{-1}) = 419 (381), 549 (2), 590 (1). HRMS (ESI) *m/z* = 670.6394 [M-Cl-H_2O+Na]$^{2+}$, 670.6353 calcd $C_{63}H_{55}GaGdNaN_{12}O_7^{2+}$. IR: 1597 cm^{-1}.

(In) porphyrin (Gd) DOTA complex 6c. The title compound was isolated as a green microcrystalline solid in 26% yield (9.3 mg, 6.56 μmol). UV-visible (MeOH): λ_{max} (nm) (ε x 10^{-3} L mol^{-1} cm^{-1}) = 418 (428), 556 (5), 595 (1). HRMS (ESI) *m/z* = 682.6372 [M-Cl-H_2O+H]$^{2+}$, 682.6332 calcd $C_{63}H_{56}GdInN_{12}O_7^{2+}$. HRMS (ESI) *m/z* = 693.6255 [M-Cl-H_2O+Na]$^{2+}$, 693.6242 calcd $C_{63}H_{55}GdInNaN_{12}O_7^{2+}$. IR: 1596 cm^{-1}.

(Mn) porphyrin (Ga) DOTA complex 6d. The title compound was isolated as a green microcrystalline solid in 76% yield (25.6 mg, 20.4 μmol). UV-visible (MeOH): λ_{max} (nm) (ε x 10^{-3} L mol^{-1} cm^{-1}) = 378 (12), 399 (12), 467 (1). HRMS (ESI) *m/z* = 1215.2964 [M-Cl]$^+$, 1215.2948 calcd $C_{63}H_{55}GaMnN_{12}O_7$. IR: 1661 cm^{-1}.

(Mn) porphyrin (Cu) NOTA complex 7a. The title compound was isolated as a green microcrystalline solid in 59% yield (18.2 mg, 15.9 μmol). UV-visible (MeOH): λ_{max} (nm) (ε x 10^{-3} L mol^{-1} cm^{-1}) = 379 (28), 411 (49), 467 (51) 565 (5), 600 (4). HRMS (ESI) *m/z* = 1109.2600 $[M\text{-}Cl]^+$, 1109.2589 calcd $C_{59}H_{49}CuMnN_{11}O_5$. IR: 1599 cm^{-1}.

(Mn) porphyrin (Ga) NOTA complex 7b. The title compound was isolated as a green microcrystalline solid in 43% yield (13.2 mg, 11.5 μmol). UV-visible (MeOH): λ_{max} (nm) (ε x 10^{-3} L mol^{-1} cm^{-1}) = 379 (31), 411 (46), 467 (57) 565 (4), 599 (4). HRMS (ESI) *m/z* = 1114.2485 $[M\text{-}Cl]^+$, 1114.2471 calcd $C_{59}H_{48}GaMnN_{11}O_5$. IR: 1600 cm^{-1}.

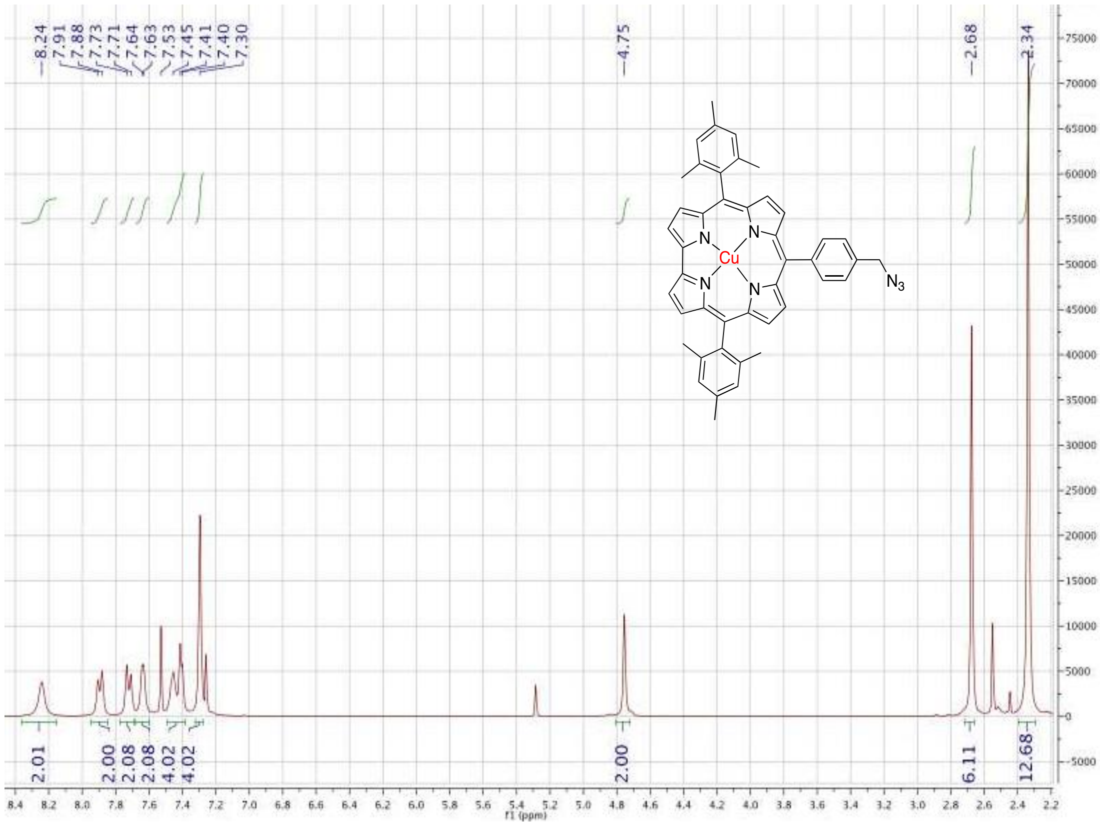

Figure S1. ^{1}H NMR spectrum of **8a** in $CDCl_3$

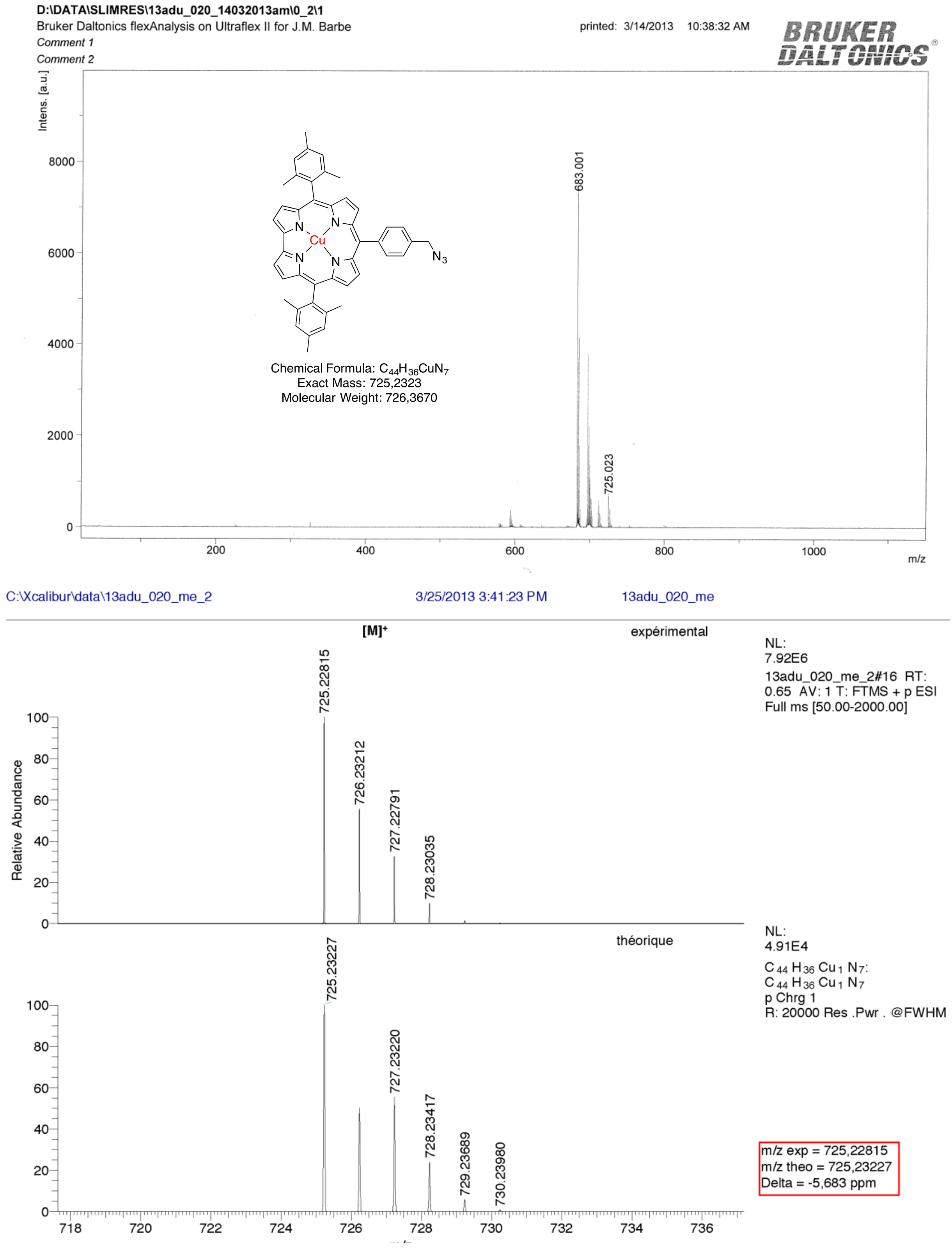

Figure S2. MS (MALDI TOF) and HRMS (ESI) mass spectra of **8a**

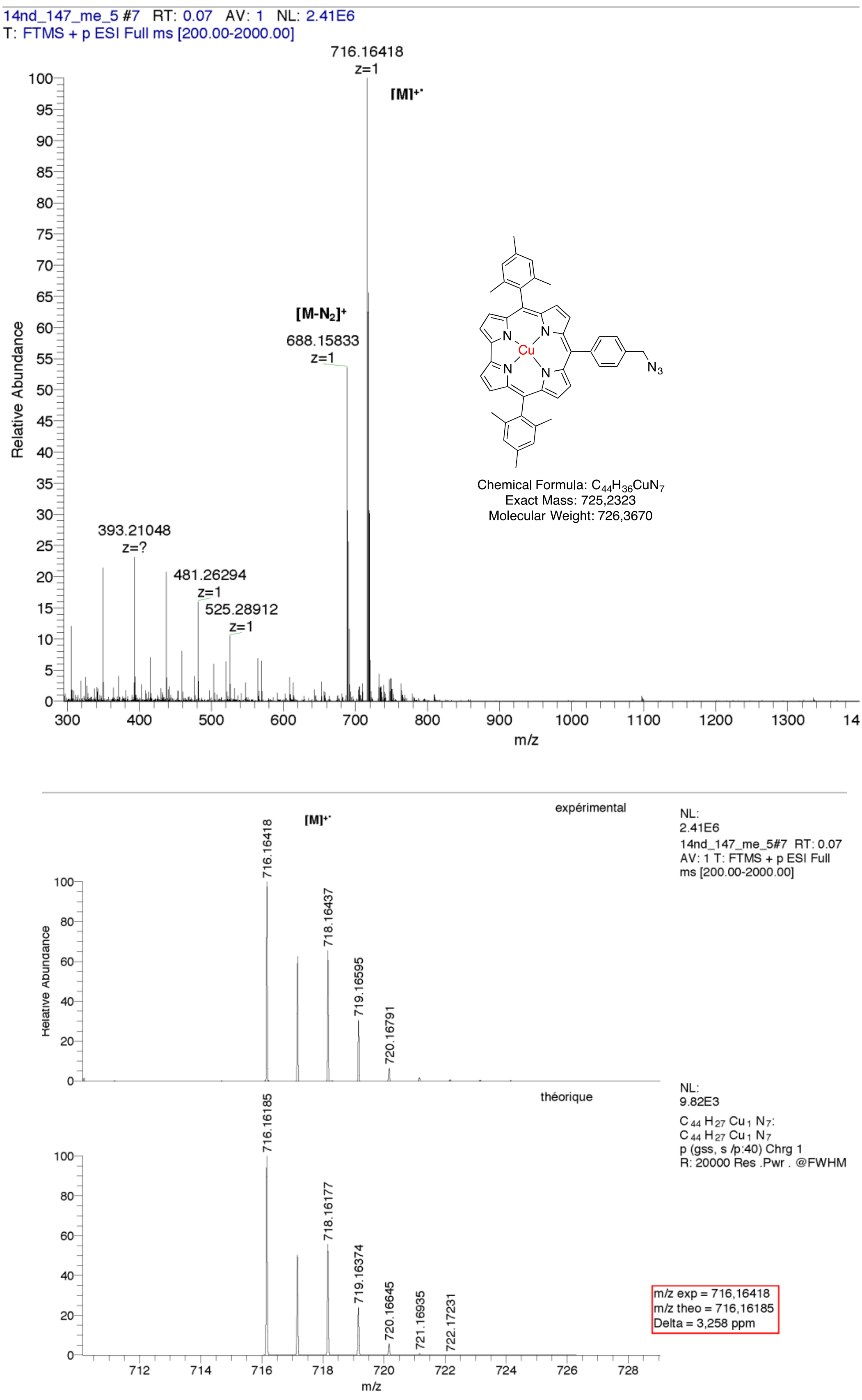

Figure S3. HRMS (ESI) mass spectrum of **9a**

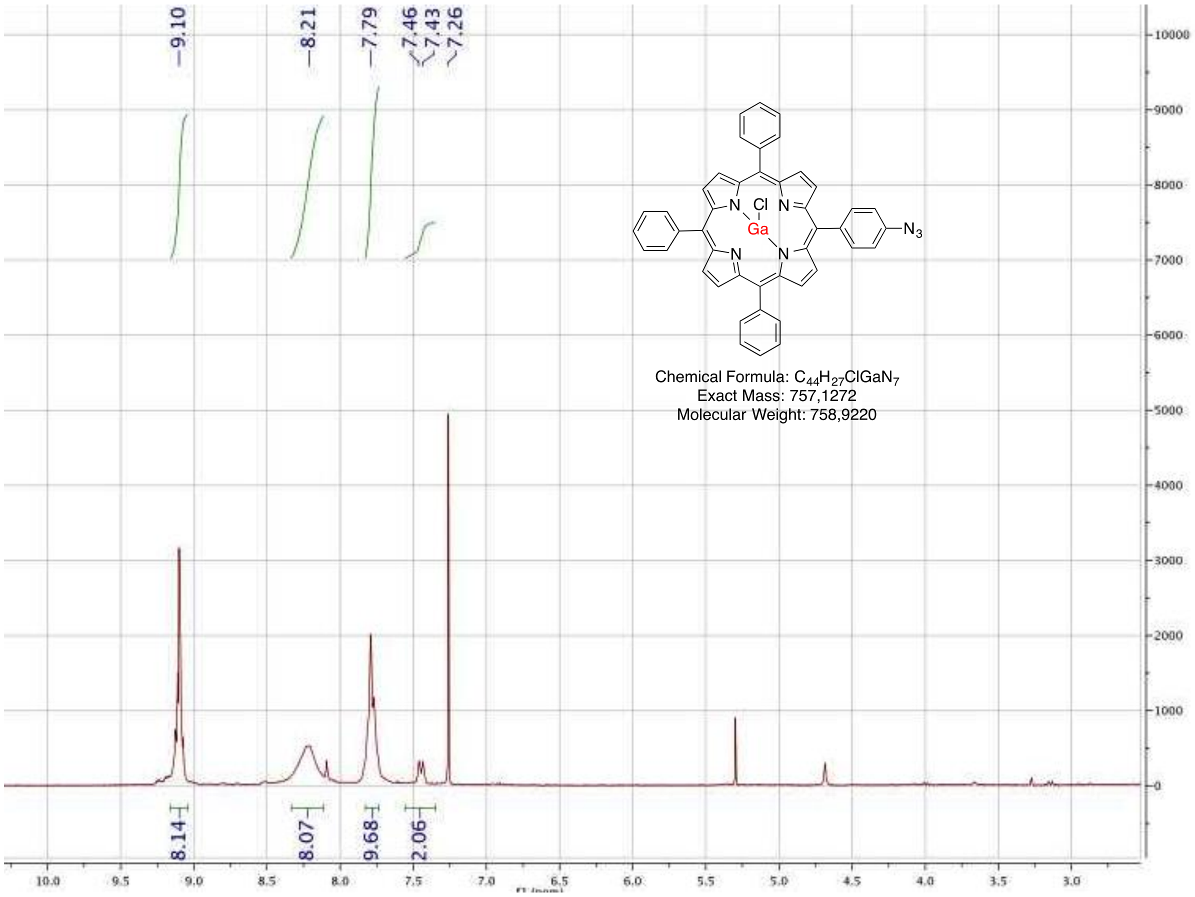

Figure S4. ^{1}H NMR spectrum of **9b** in $CDCl_3$

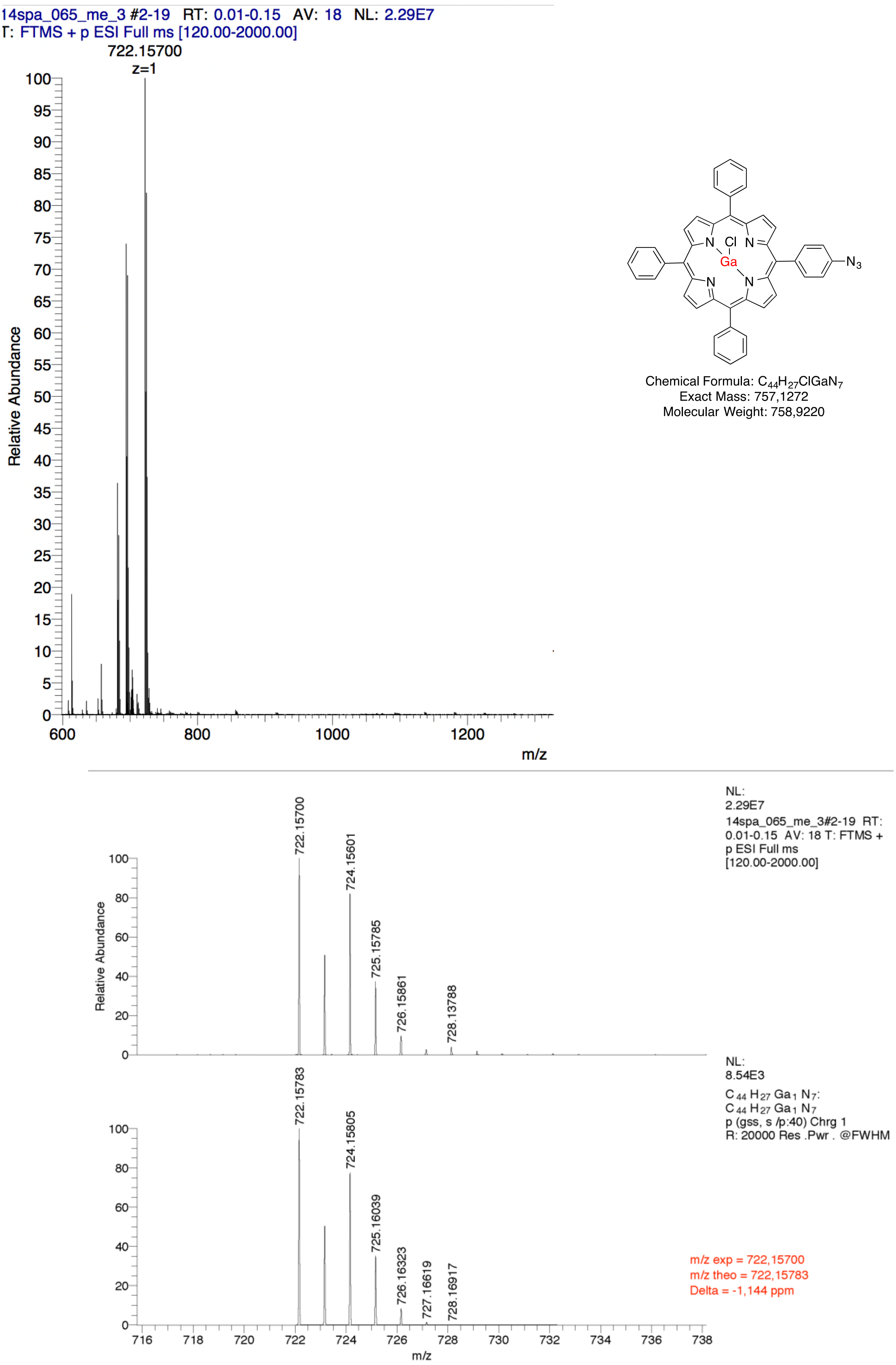

Figure S5. HRMS (ESI) mass spectrum of **9b**

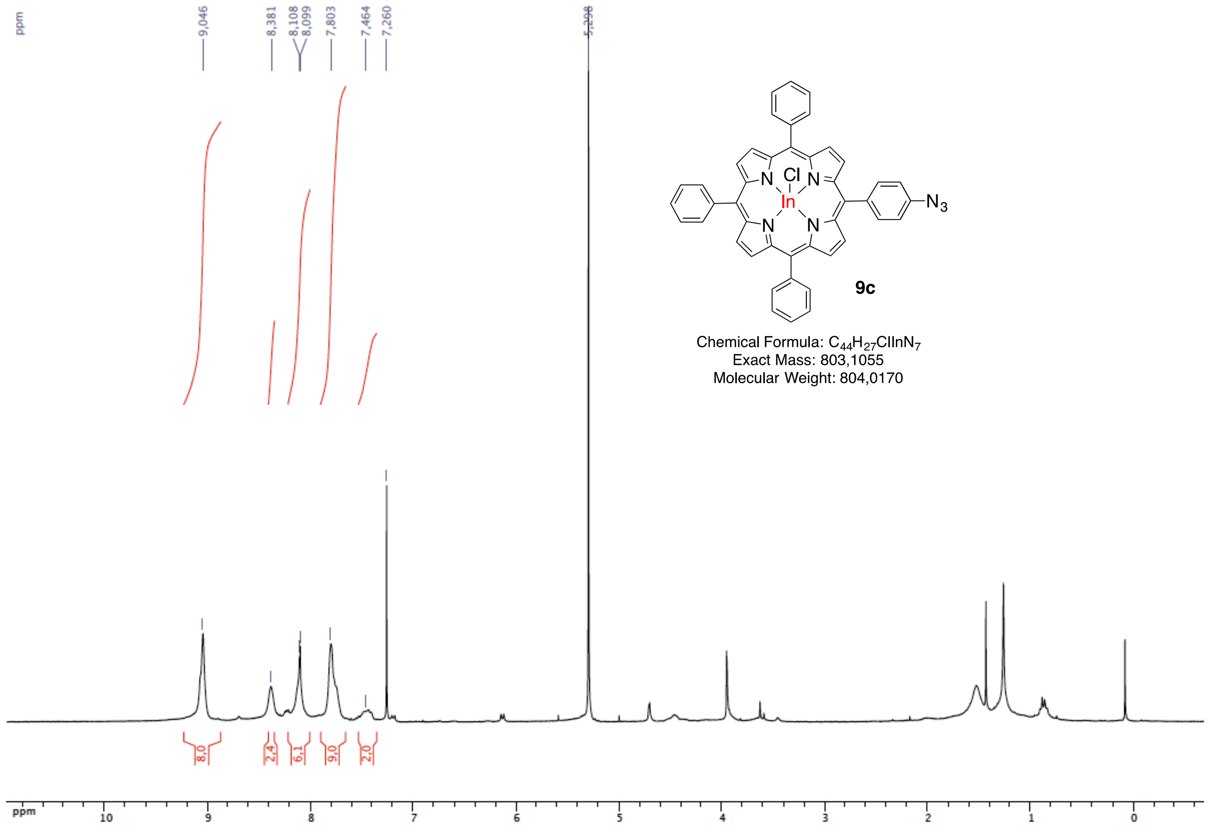

Figure S6. ¹H NMR spectrum of **9c** in $CDCl_3$

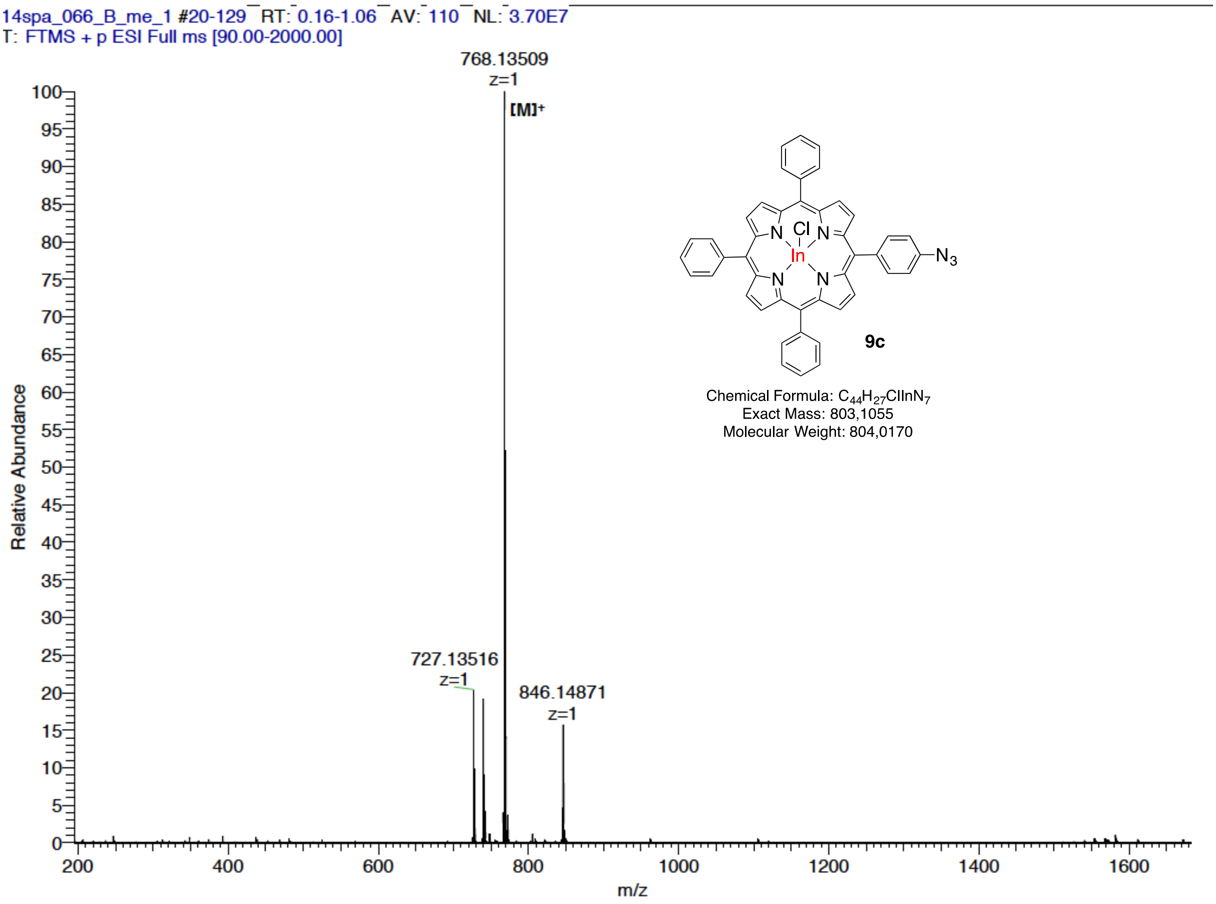

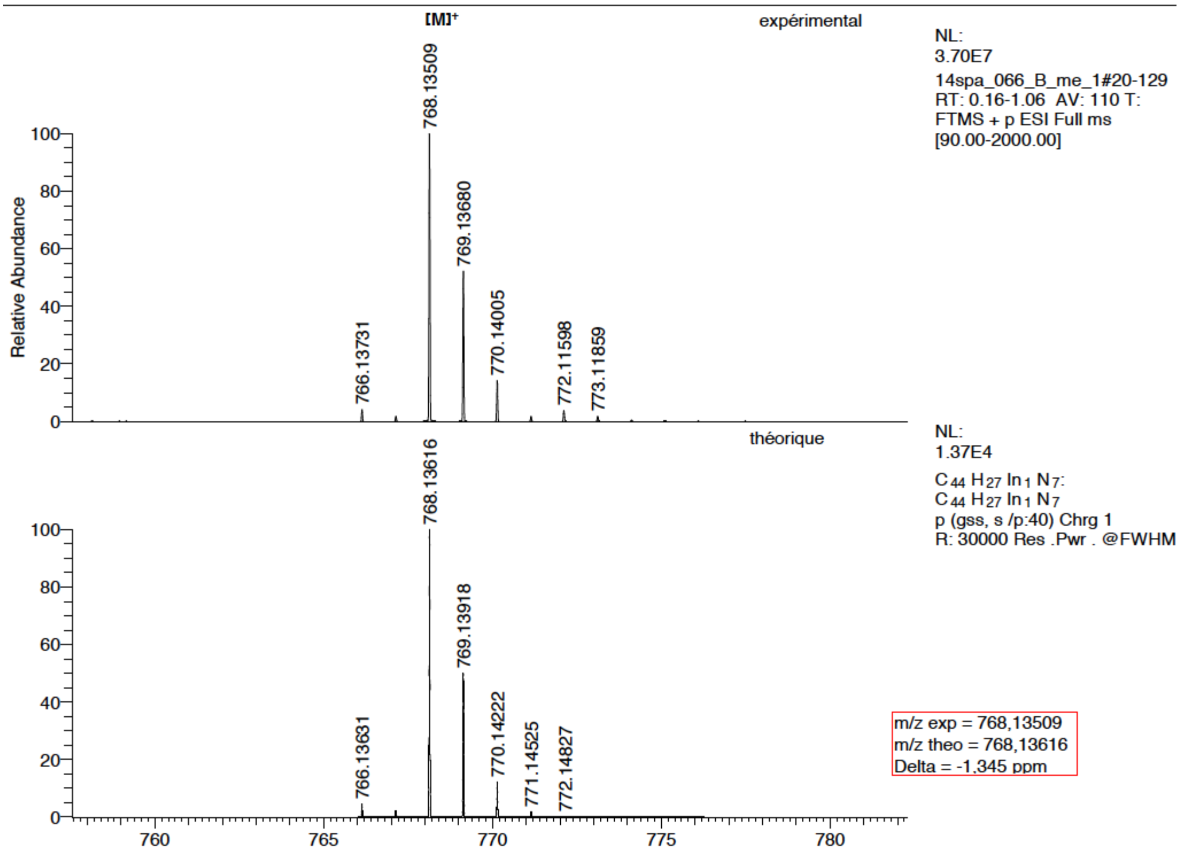

Figure S7. HRMS (ESI) mass spectrum of **9c**

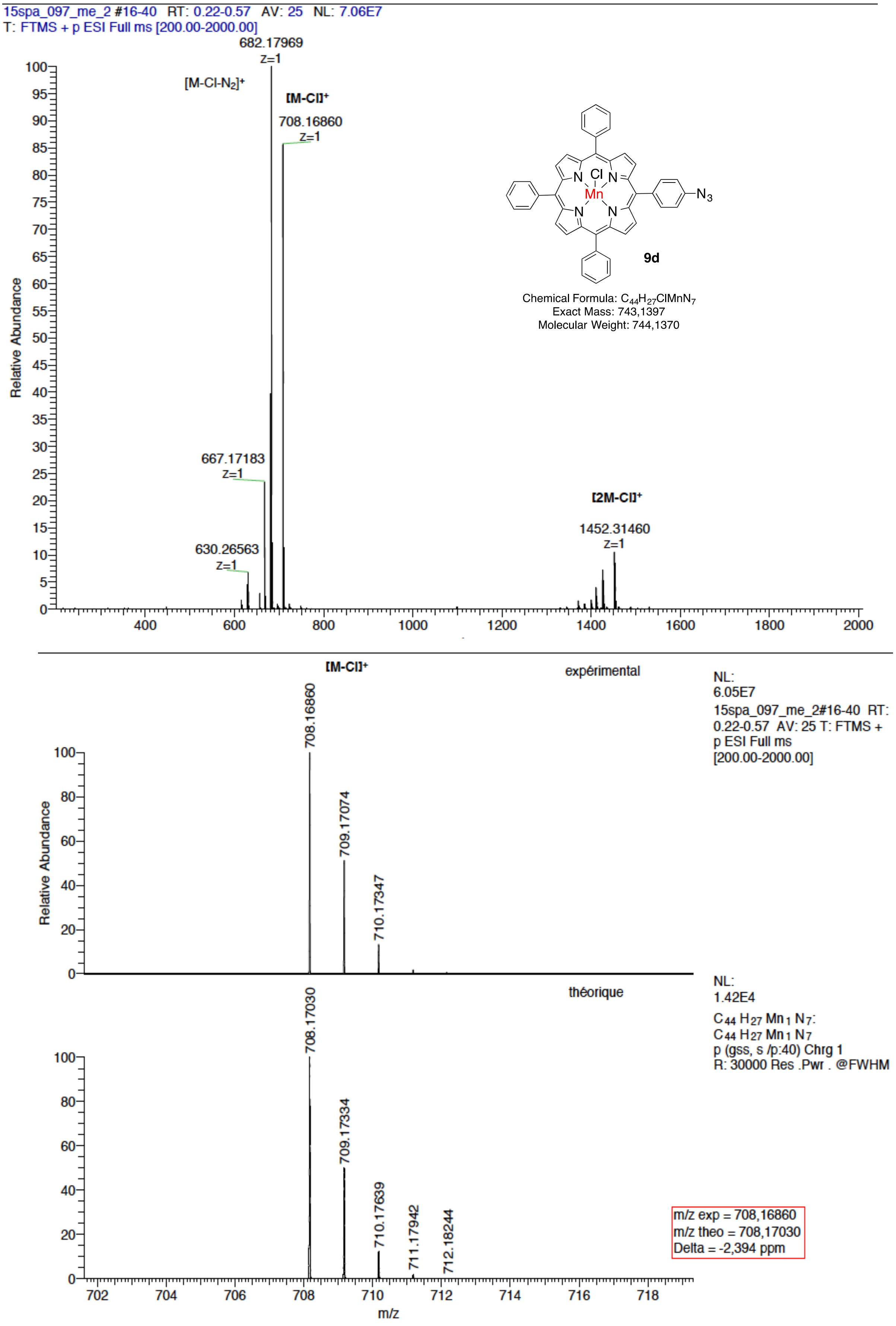

Figure S8. HRMS (ESI) mass spectrum of **9d**

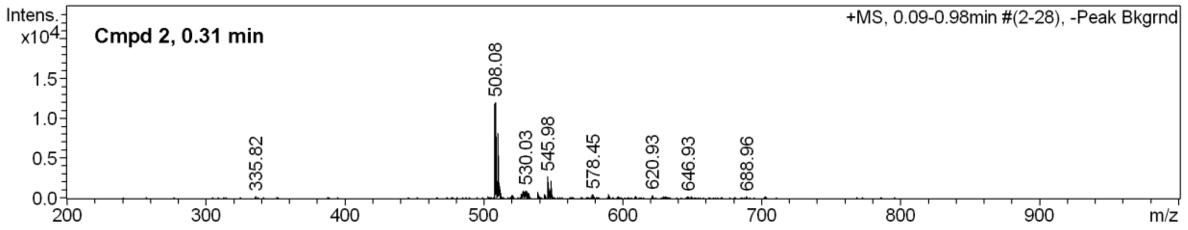

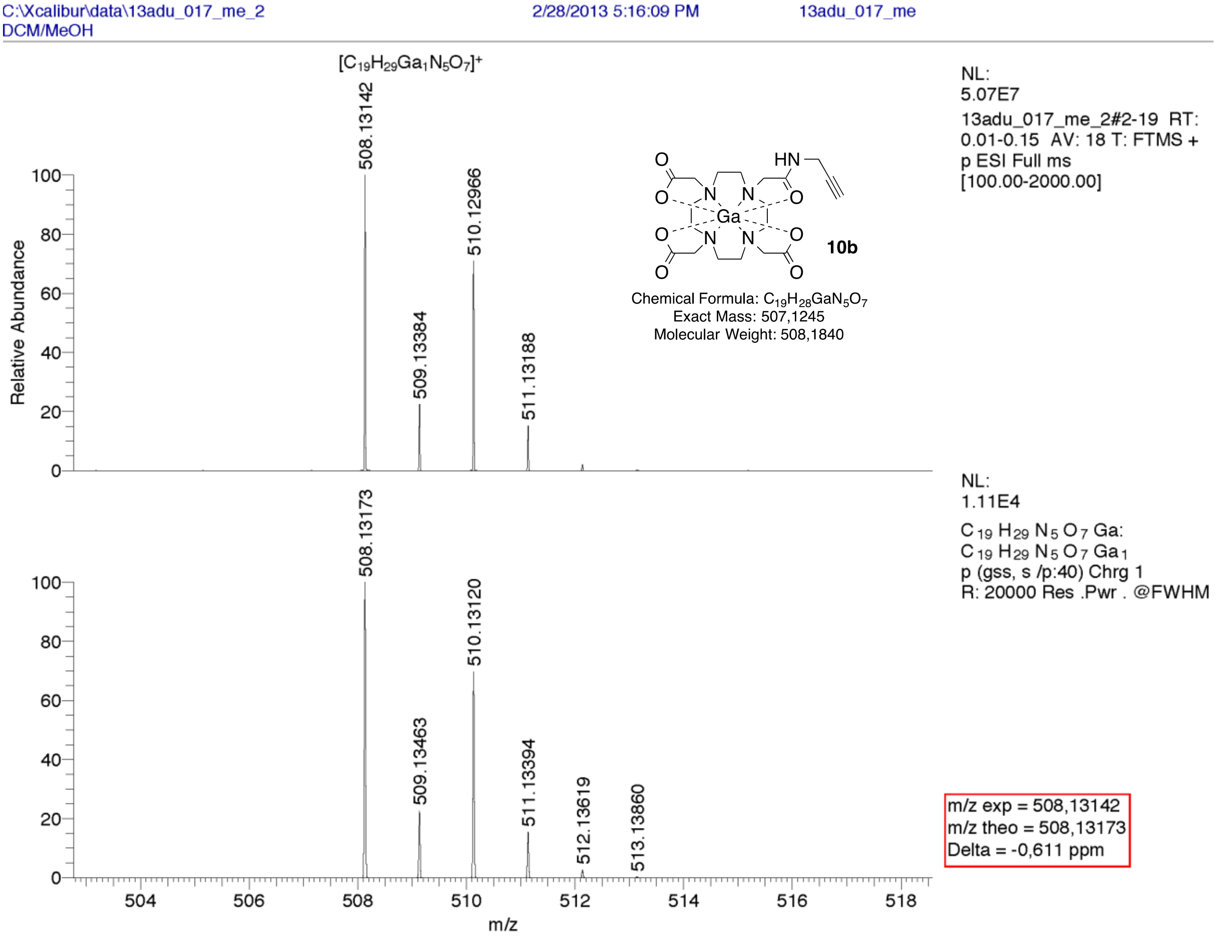

Figure S9. MS and HRMS (ESI) mass spectra of **10b**

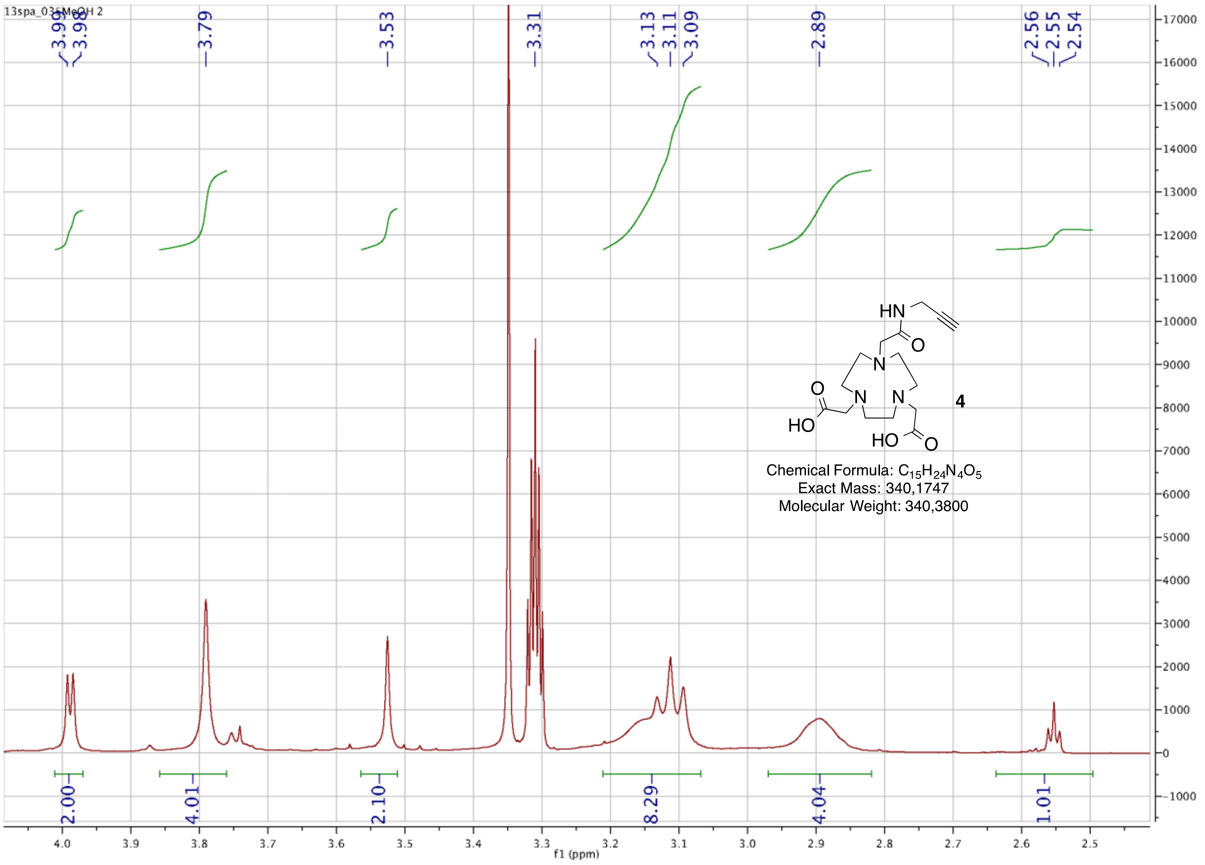

Figure S10. ^{1}H NMR spectrum of **4** in MeOD

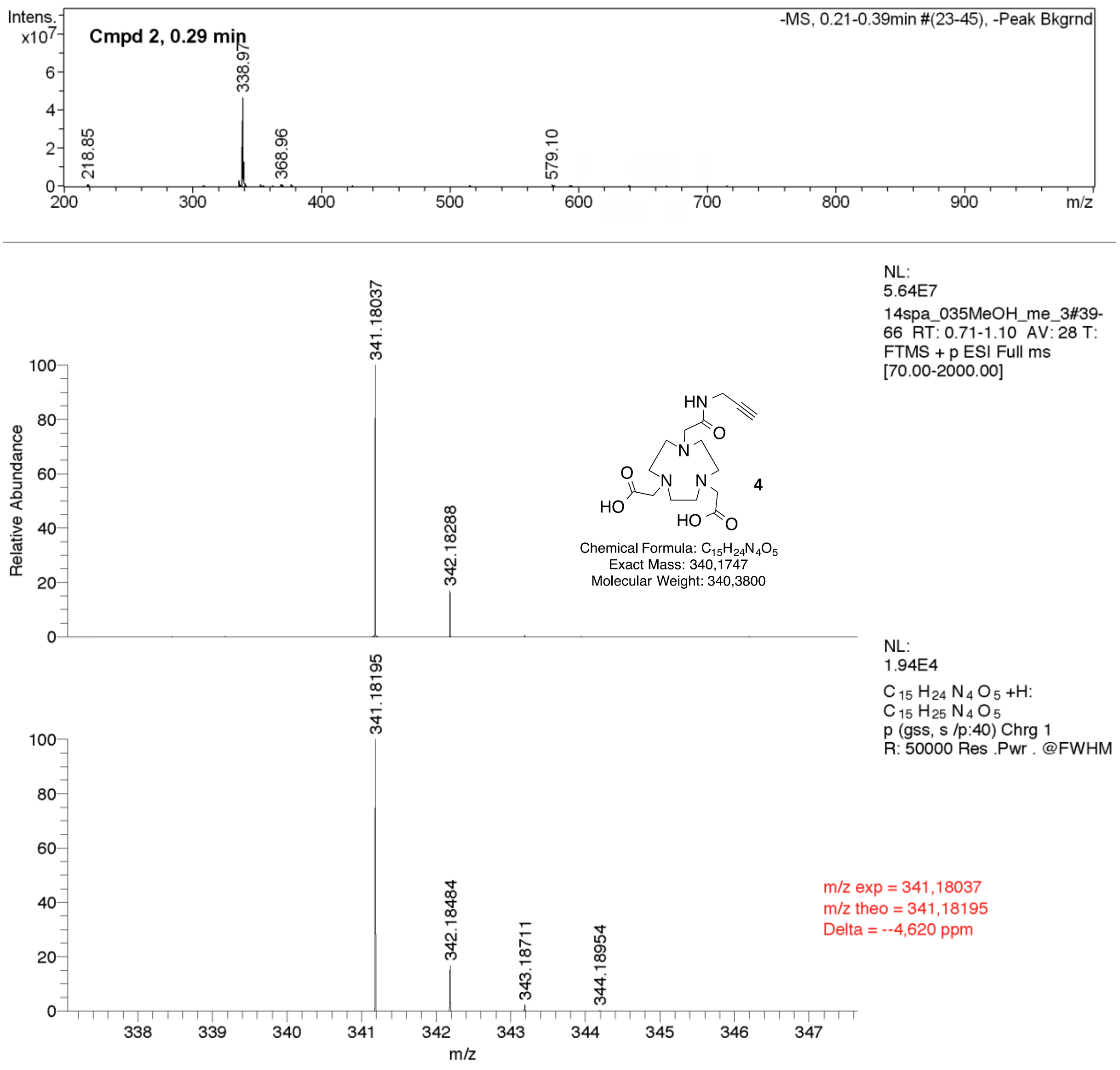

Figure S11. MS and HRMS (ESI) mass spectra of **4**

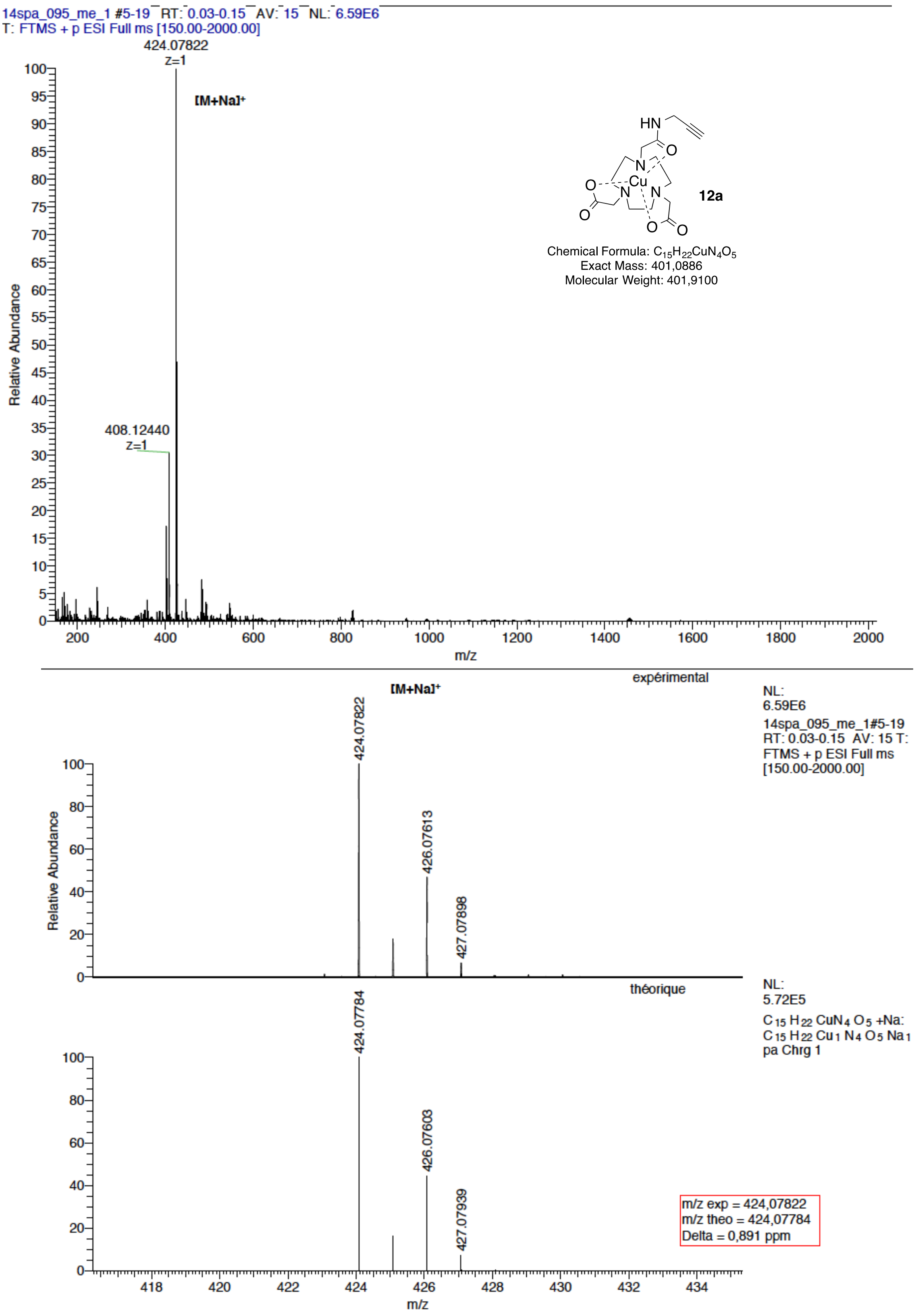

Figure S12. HRMS (ESI) mass spectrum of **12a**

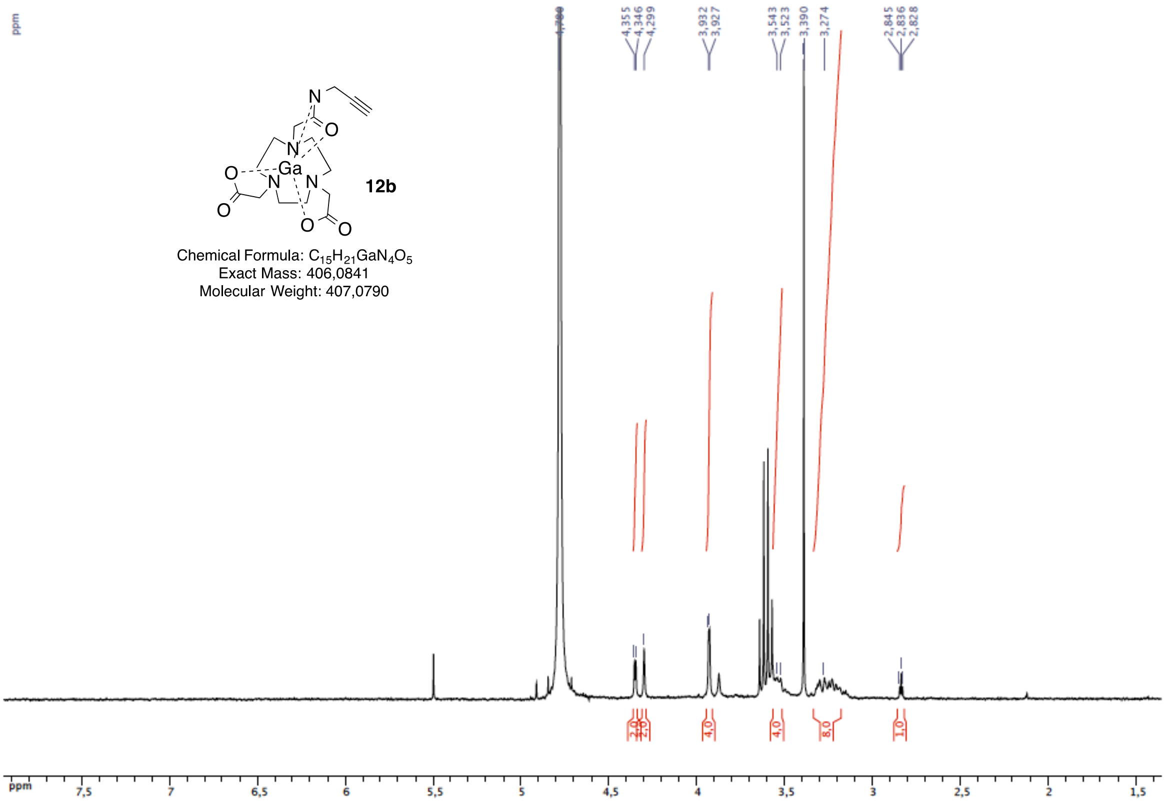

Figure S13. ^{1}H NMR spectrum of **12b** in D_2O

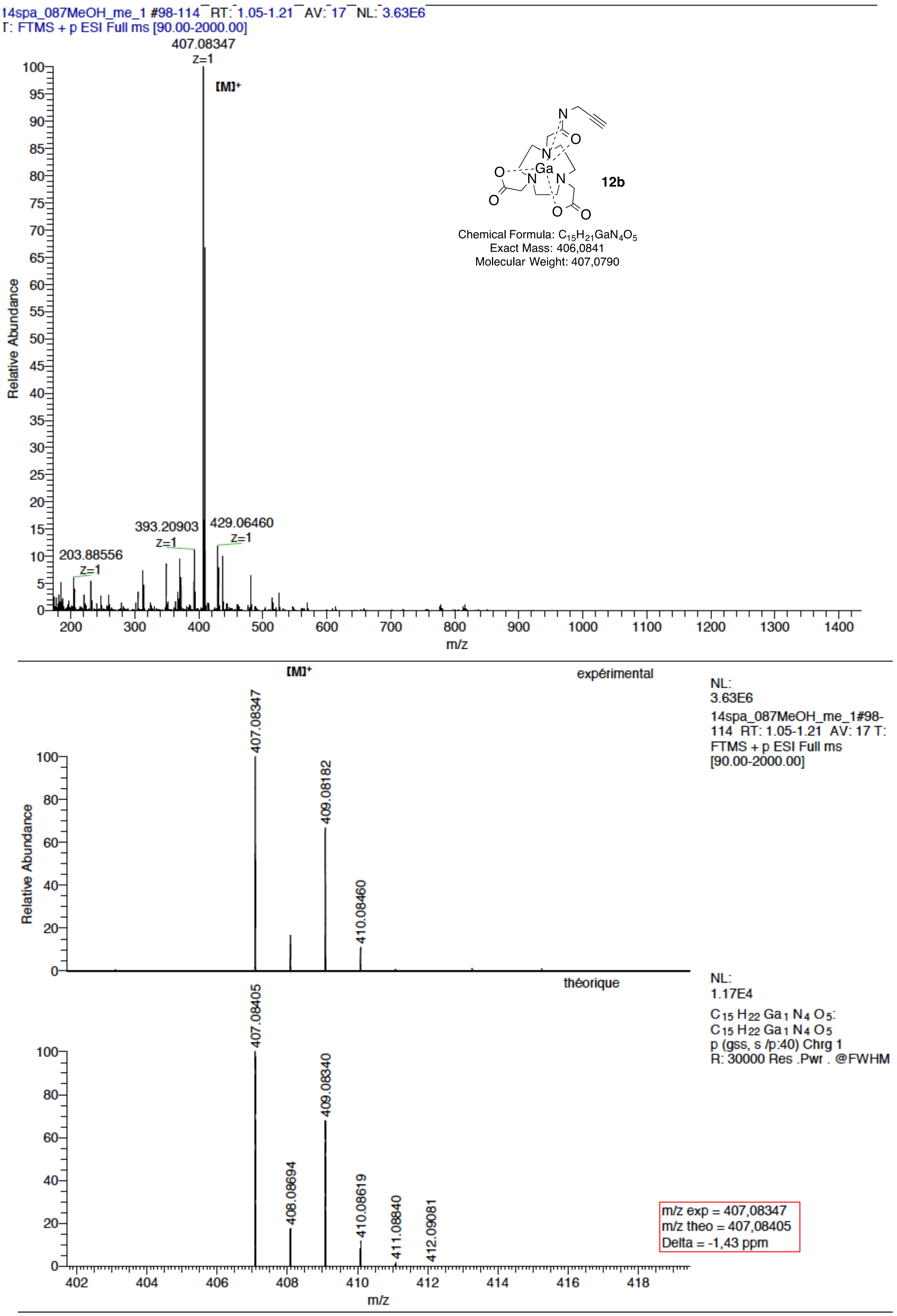

Figure S14. HRMS (ESI) mass spectrum of **12b**

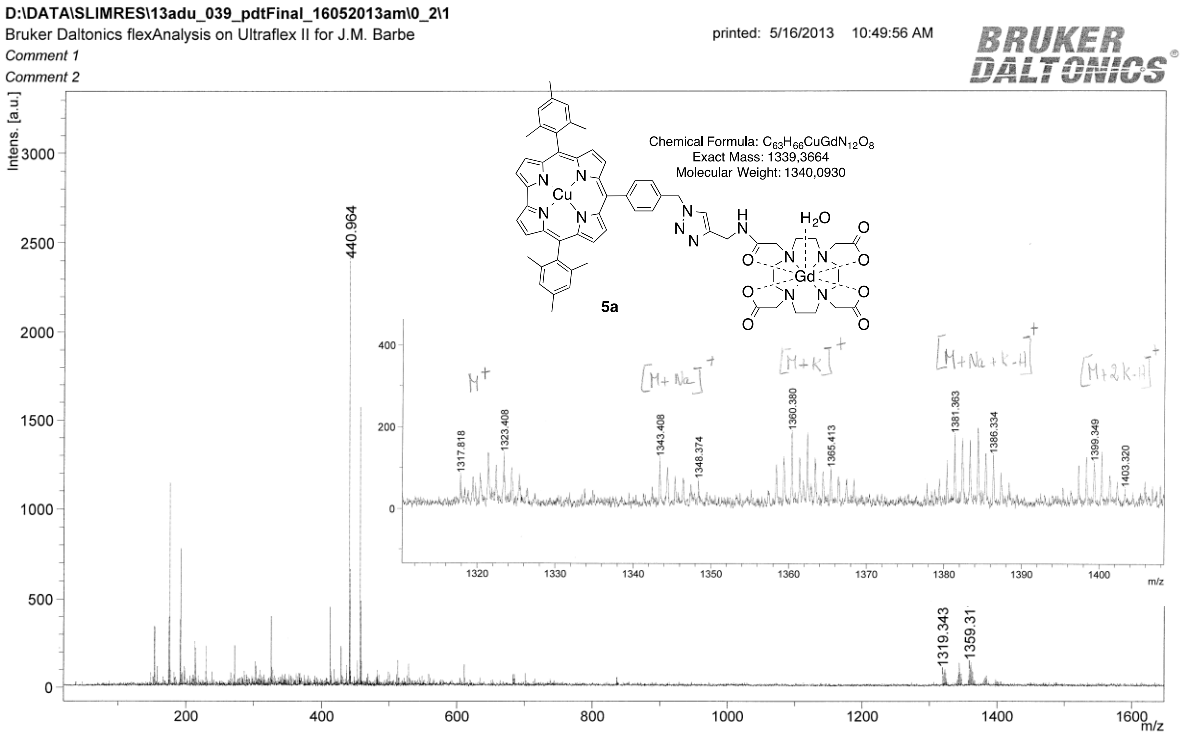

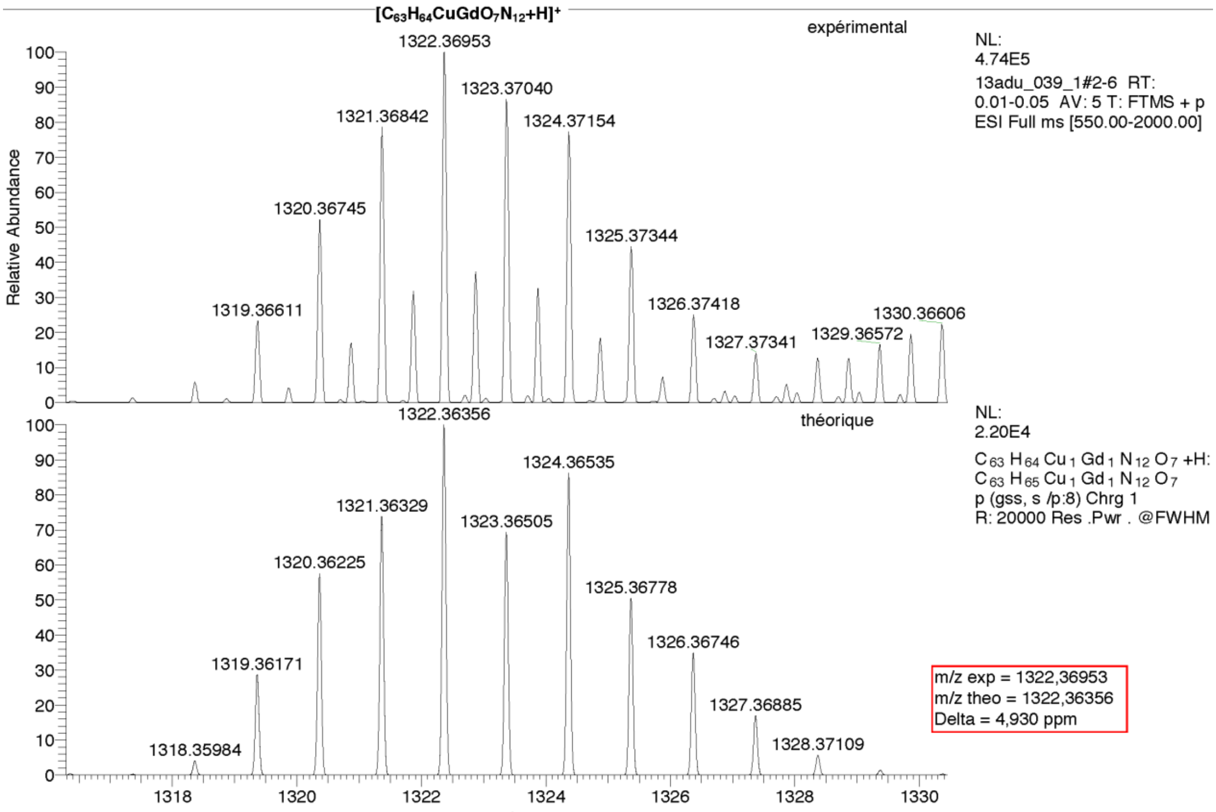

Figure S15. MS (MALDI TOF) and HRMS (ESI) mass spectra of **5a**

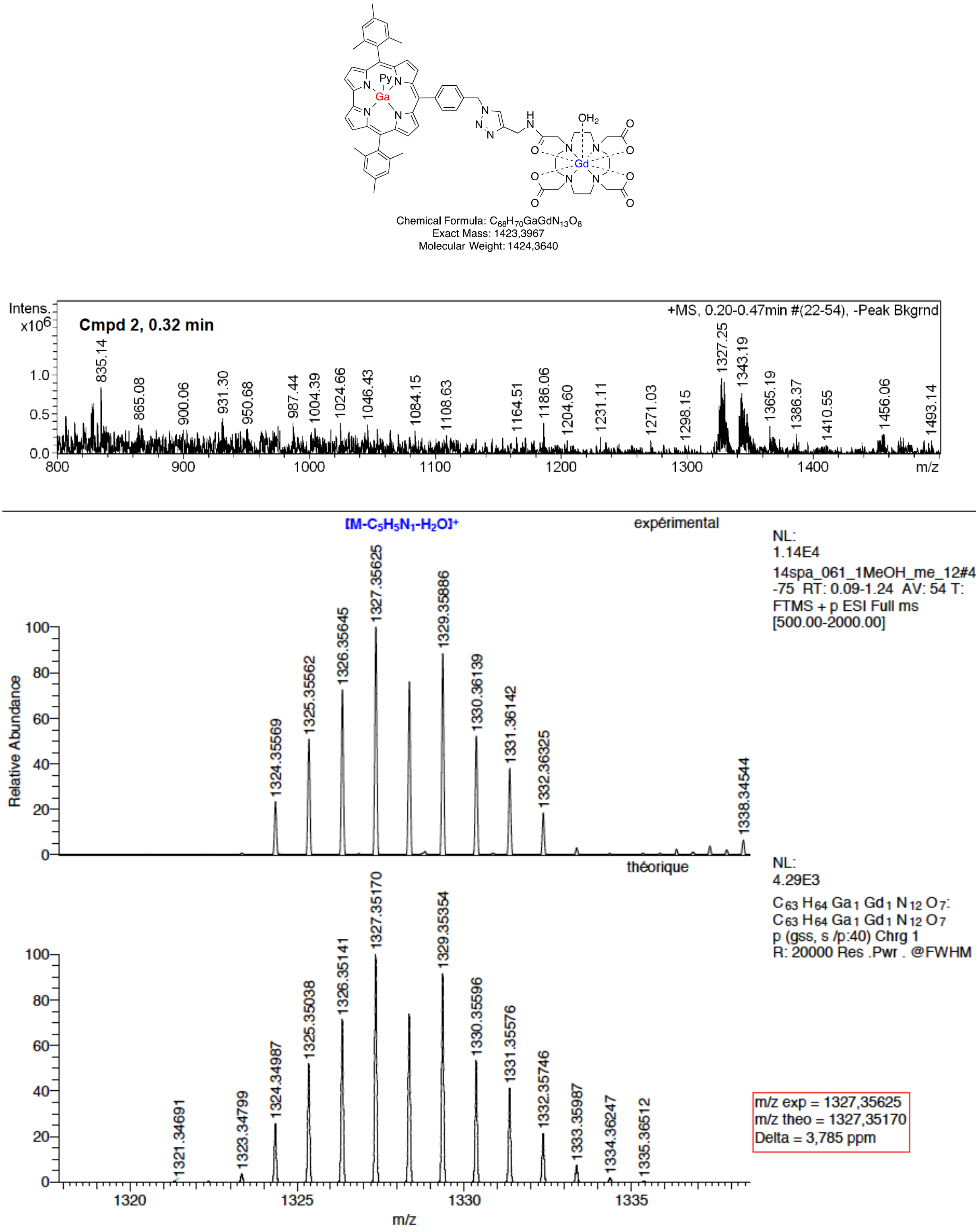

Figure S16. MS and HRMS (ESI) mass spectra of **5b**

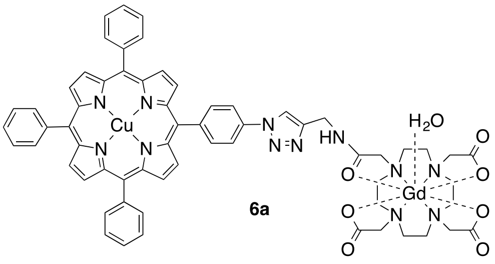

6a

Chemical Formula: $C_{63}H_{57}CuGdN_{12}O_8$
Exact Mass: 1330,2959
Molecular Weight: 1331,0210

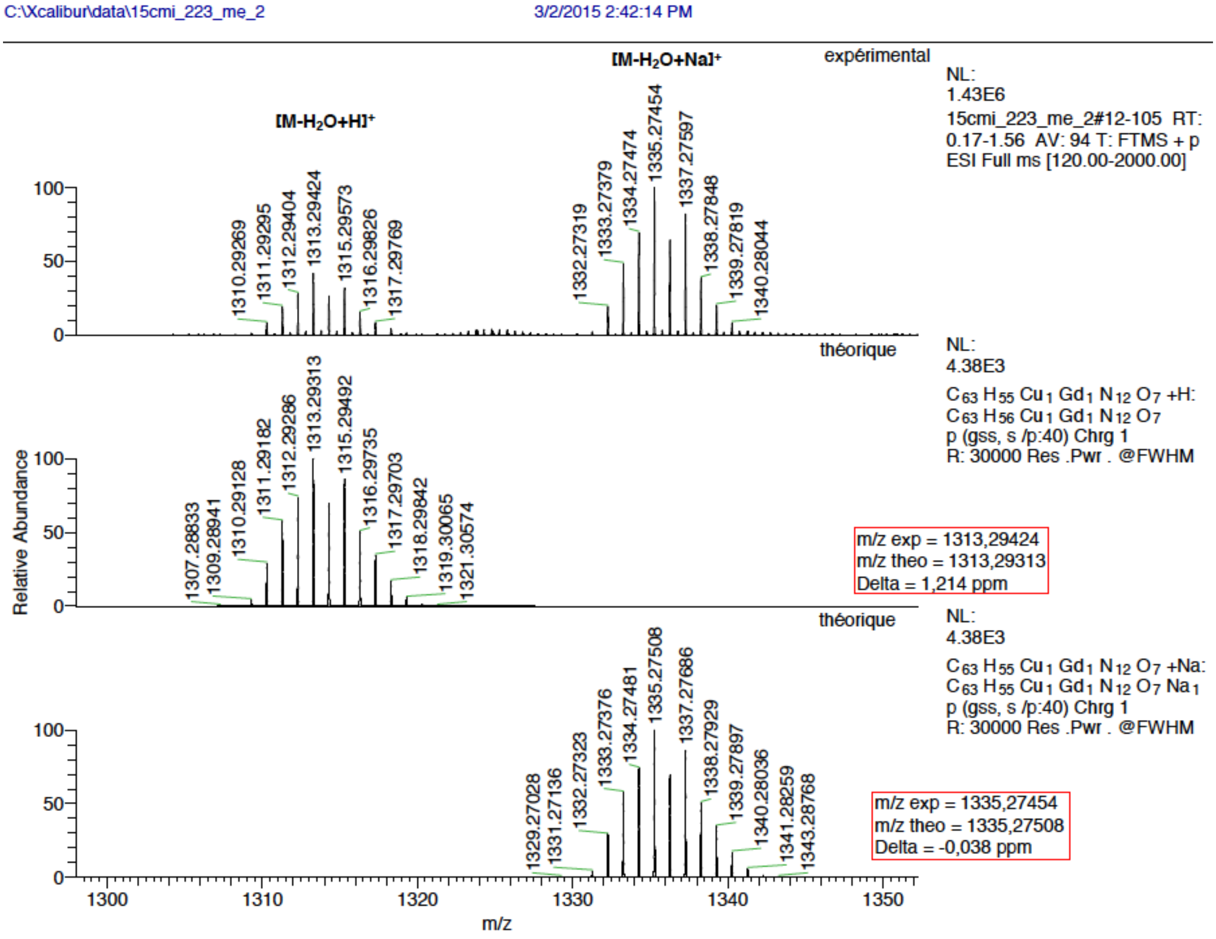

Figure S17. HRMS (ESI) mass spectrum of **6a**

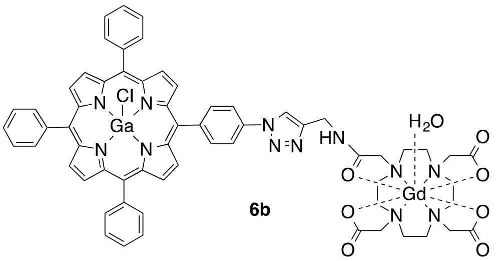

Chemical Formula: $C_{63}H_{57}ClGaGdN_{12}O_8$
Exact Mass: 1371,2608
Molecular Weight: 1372,6480

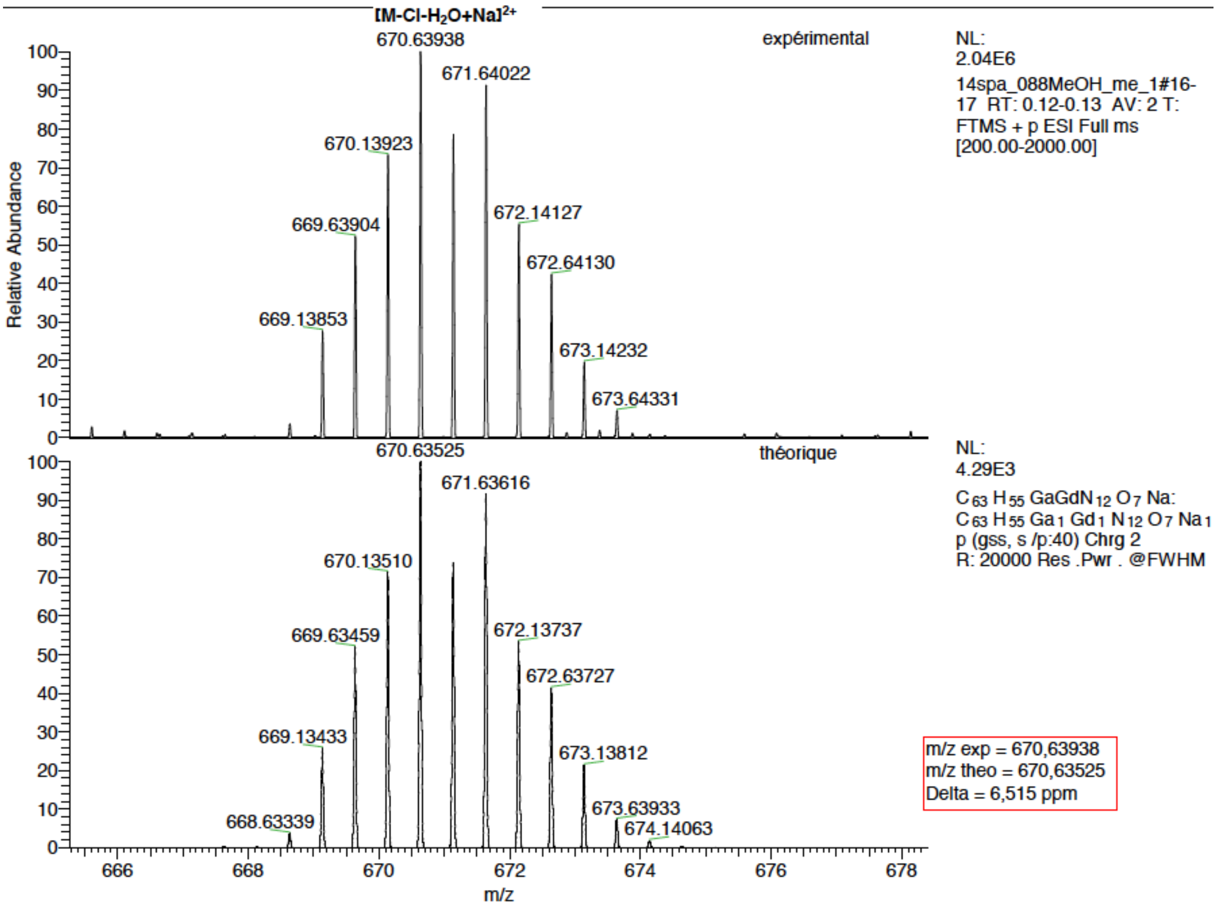

Figure S18. HRMS (ESI) mass spectrum of **6b**

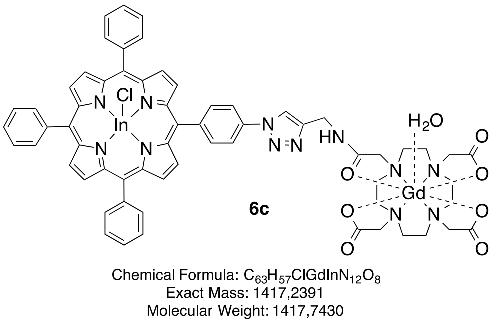

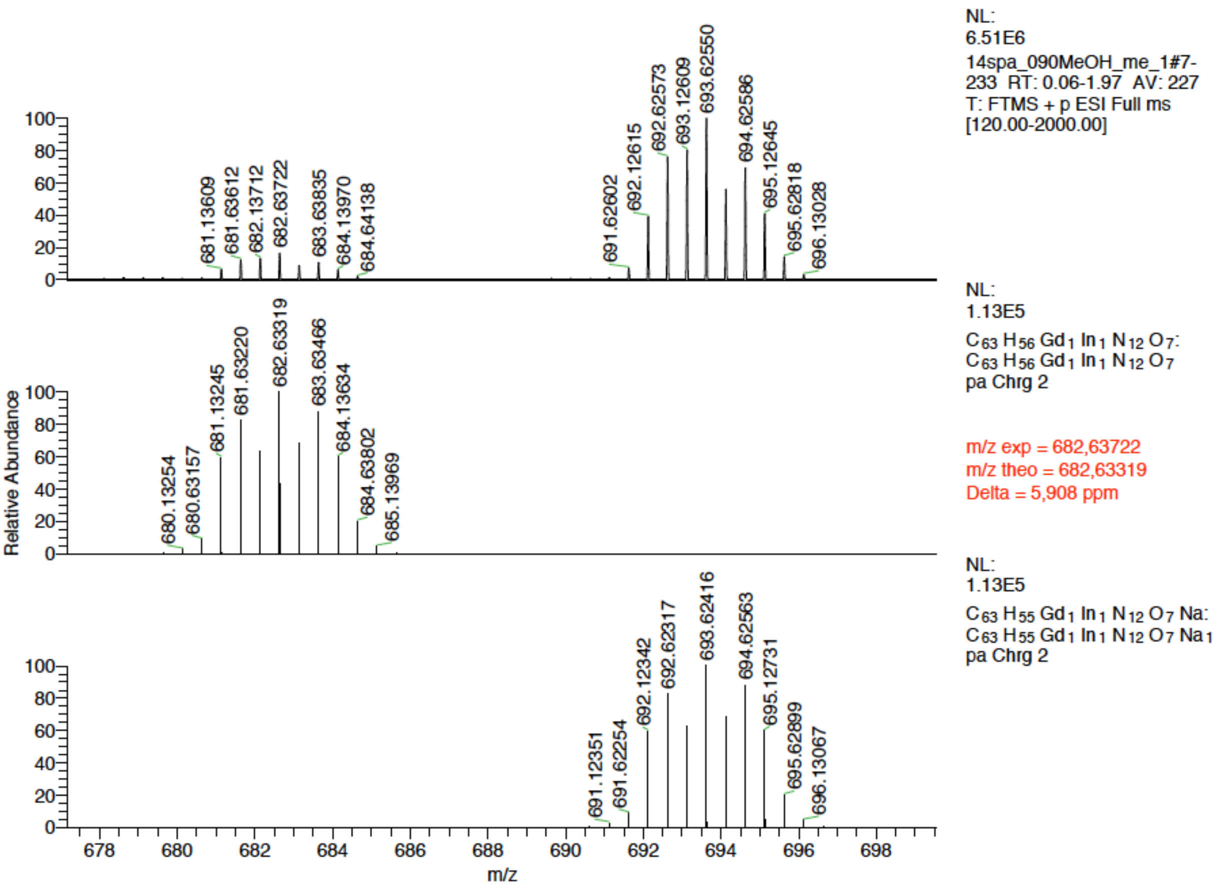

Figure S19. HRMS (ESI) mass spectrum of **6c**

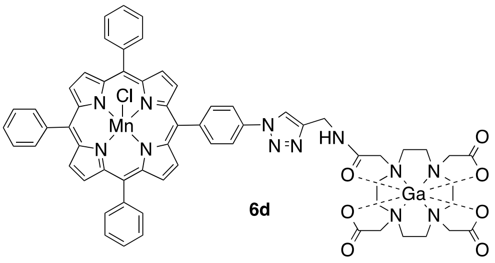

Chemical Formula: $C_{63}H_{55}ClGaMnN_{12}O_7$
Exact Mass: 1250,2642
Molecular Weight: 1252,3210

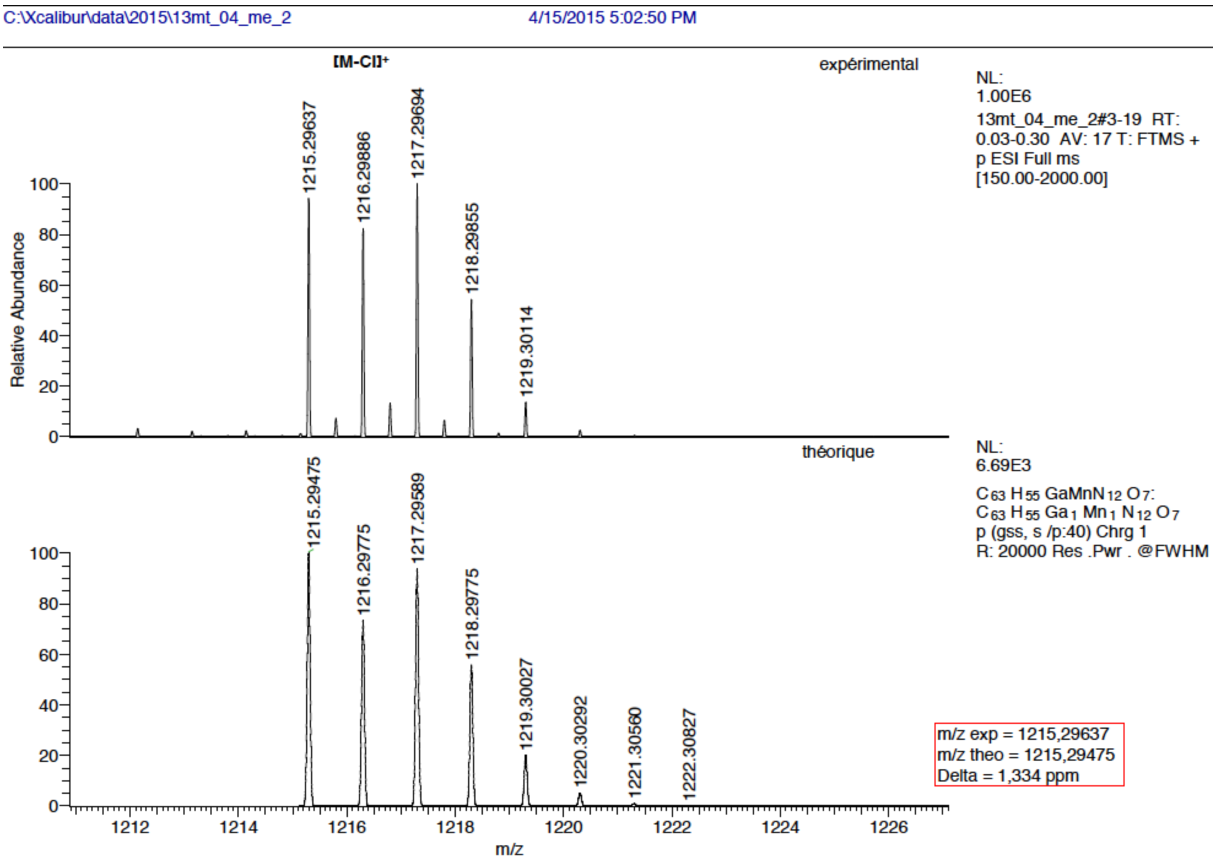

Figure S20. HRMS (ESI) mass spectrum of **6d**

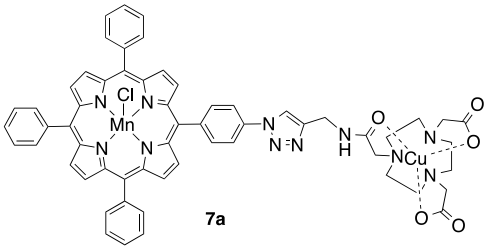

Chemical Formula: $C_{59}H_{49}ClCuMnN_{11}O_5$
Exact Mass: 1144,2283
Molecular Weight: 1146,0470

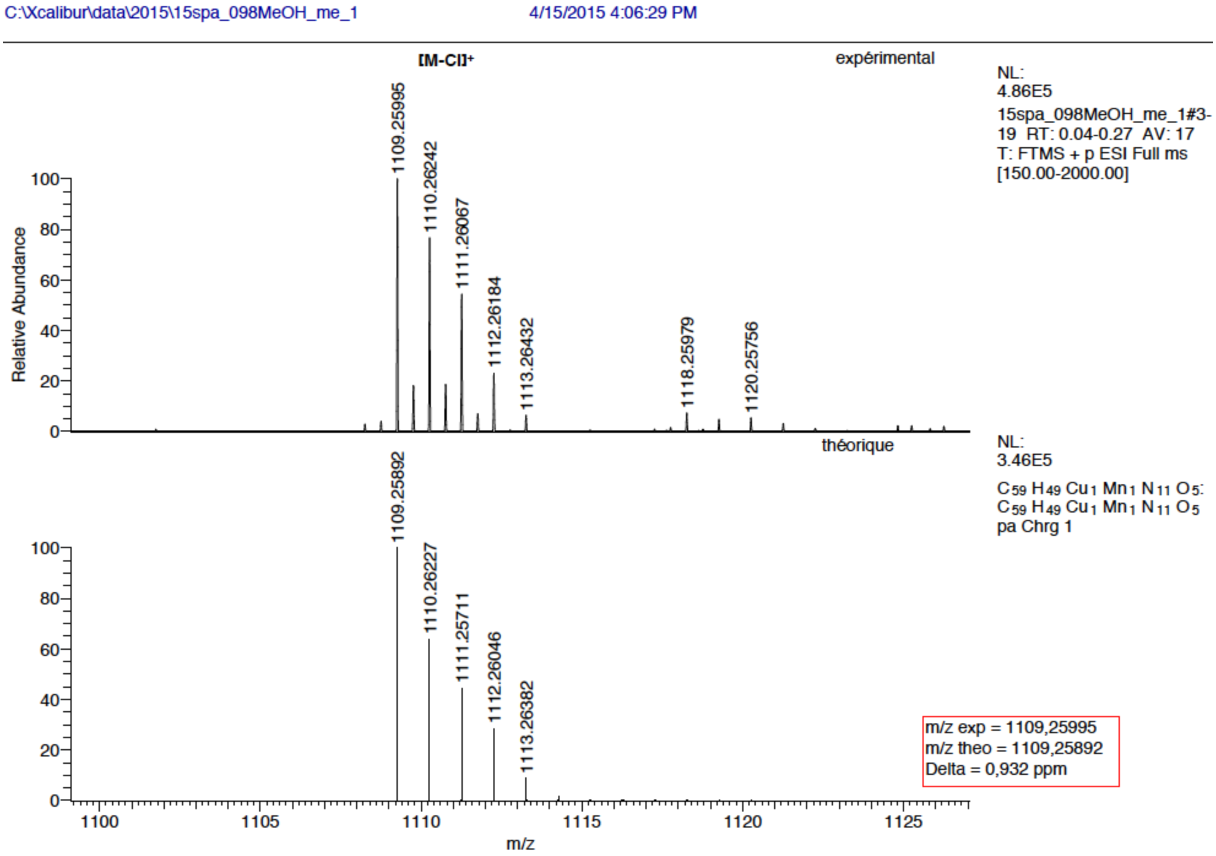

Figure S21. HRMS (ESI) mass spectrum of **7a**

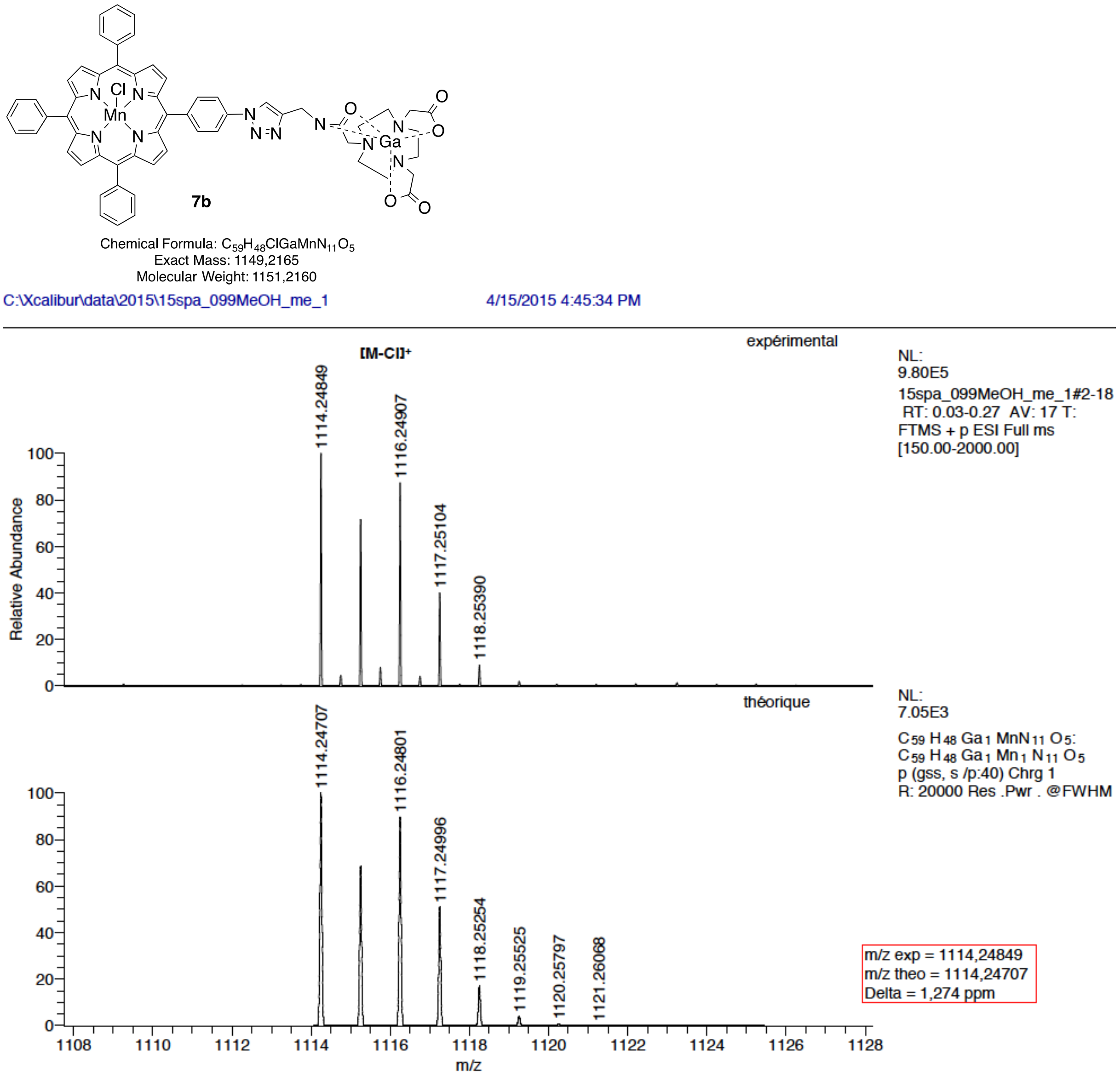

Figure S22. HRMS (ESI) mass spectrum of **7b**

References:

1. Brizet, B.; Desbois, N.; Bonnot, A.; Langlois, A.; Dubois, A.; Barbe, J. M.; Gros, C. P.; Goze, C.; Denat, F.; Harvey, P. D., *Inorg. Chem.* **2014**, *53*, 3392-3403.
2. Fernandez-Trillo, F.; Pacheco-Torres, J.; Correa, J.; Ballesteros, P.; Lopez-Larrubia, P.; Cerdan, S.; Riguera, R.; Fernandez-Megia, E., *Biomacromolecules* **2011**, *12*, 2902-2907.
